# Intraspecific competition counters the effects of elevated and optimal temperatures on phloem-feeding insects in tropical and temperate rice

**DOI:** 10.1371/journal.pone.0240130

**Published:** 2020-10-06

**Authors:** Finbarr G. Horgan, Arriza Arida, Goli Ardestani, Maria Liberty P. Almazan

**Affiliations:** 1 EcoLaVerna Integral Restoration Ecology, Kildinan, Co. Cork, Ireland; 2 Environment and Sustainable Resource Management, University College Dublin, Belfield, Dublin, Ireland; 3 International Rice Research Institute, Makati, Metro Manila, Philippines; 4 Department of Veterinary and Animal Sciences, University of Massachusetts, Amherst, Massachusetts, United States of America; USDA Agricultural Research Service, UNITED STATES

## Abstract

The direct effects of rising global temperatures on insect herbivores could increase damage to cereal crops. However, the indirect effects of interactions between herbivores and their biotic environment at the same temperatures will potentially counter such direct effects. This study examines the potential for intraspecific competition to dampen the effects of optimal temperatures on fitness (survival × reproduction) of the brown planthopper, *Nilaparvata lugens* [BPH] and whitebacked planthopper, *Sogatella furcifera* [WBPH], two phloem-feeders that attack rice in Asia. We conducted a series of experiments with increasing densities of ovipositing females and developing nymphs on tropical and temperate rice varieties at 25, 30 and 35°C. Damage from planthoppers to the tropical variety was greater at 30°C compared to 25°C, despite faster plant growth rates at 30°C. Damage to the temperate variety from WBPH nymphs was greatest at 25°C. BPH nymphs gained greater biomass at 25°C than at 30°C despite faster development at the higher temperature (temperature-size rule); however, the effect was apparent only at high nymph densities. WBPH survival, development rates and nymph weights all declined at ≥ 30°C. At about the optimal temperature for WBPH (25°C), intraspecific crowding reduced nymph weights. Temperature has little effect on oviposition responses to density, and intraspecific competition between females only weakly counters the effects of optimal temperatures on oviposition in both BPH and WBPH. Meanwhile, the deleterious effects of nymph crowding will counter the direct effects of optimal temperatures on voltinism in BPH and on body size in both BPH and WBPH. The negative effects of crowding on BPH nymphs may be decoupled from resource use at higher temperatures.

## Introduction

Global temperatures have increased by between 0.5 and 0.9°C since the 1960s and are predicted to continue rising under current global CO_2_ emissions [1, 2]. Warmer temperatures are associated with an increasing diversity of insect herbivores at higher latitudes and altitudes as insect distribution ranges shift poleward and peak-ward [3, 4]. Warmer temperatures will also directly affect insect herbivores by increasing the numbers of generations they achieve in a single season or crop cycle [5, 6]. These changes are predicted to increase herbivore damage to plants, including crops [7–10]. However, evidence of increased damage to crops from insect herbivores under warmer climates is rare and often anecdotal, or suffers from the problems of ‘cause-and-effect’ associated with correlative studies [1, 7, 11]. Furthermore, several studies have indicated that, whereas higher temperatures can have direct positive effects on many insect herbivores by providing optimal temperatures for growth and development, the indirect negative effects of elevated temperatures–mediated through herbivore interactions with their biotic environment–can sometimes dampen the impacts of a warming climate [12–14]. For example, the natural enemies of herbivore pests may have greater attack efficiency at higher temperatures, or may increase the numbers of generations they achieve in a season to a greater extent than observed among their prey [3, 15].

Some recent studies have suggested that intra- and interspecific competition can also dampen the effects of higher temperatures on herbivore development and on the damage herbivores cause to crops and other plants [14, 16, 17]. At optimal temperatures, insect herbivores will develop faster, increase their feeding rates and body mass, and produce more eggs [1, 18]. Meanwhile, intraspecific competition can delay herbivore development and/or reduce body mass and fecundity [19, 20], which would counter the direct positive effects of optimal temperatures. However, because ambient temperatures affect both the development of herbivores and the growth of their host plants [13, 21, 22], the potential negative impacts of intraspecific crowding at optimal and elevated temperatures might not always translate into positive effects on the plant host. Intuitively, the outcome of these interactions will depend on the densities at which intraspecific competition becomes detrimental for the insects and on the capacity of the host plant to tolerate herbivore damage, both of which are ultimately influenced by resource availability, including optimal temperatures [16, 19, 20, 23, 24].

The brown planthopper (BPH), *Nilaparvata lugens*, and the whitebacked planthopper (WBPH), *Sogatella furcifera*, co-occur as pests of rice, *Oryza sativa*, in Asia [25, 26]. BPH is often considered as the principal pest of rice throughout the region. Outbreaks of BPH are associated with intensive rice production in irrigated lowlands where farmers apply high concentrations of nitrogenous fertilizers and resurgence insecticides [26, 27]. WBPH is a major pest of hybrid rice varieties that has become more prominent throughout Asia in recent decades [18, 25]. Increased damage to rice from both planthoppers has been associated with elevated temperatures, particularly since the early 2000s [28–30]. Research indicates that the species occupy different thermal niches: optimal temperatures for nymph survival, nymph growth and oviposition are about 5°C higher for BPH than for WBPH [18]. Because they undergo long distance migrations during the northern hemisphere spring, the species will occur in both tropical and temperate rice fields where they are exposed to relatively hot-humid or cool-dry conditions, respectively. Whereas BPH and WBPH have relatively conserved temperature reaction norms over their distribution ranges [18], tropical and temperate rice varieties both display a wide range of temperature optima [31–33]. Therefore, the damage that planthoppers cause to rice plants is expected to vary depending on the relative rates of rice growth and of insect development under local climatic conditions.

In the present study, we examine the effects of temperature on intraspecific competition in BPH and WBPH and examine the relative impacts of temperature and herbivore density on damage to their rice hosts. In our experiments, we varied planthopper adult and nymph densities on tropical and temperate rice plants in environmental chambers and assessed planthopper fitness (survival × reproduction) and relative damage to the plants. The lowest temperature used in our experiments (25°C) was about optimal for WBPH oviposition and development. Higher temperatures were included among the optimal temperatures for BPH (i.e., 25–30°C) or were close to the critical upper temperature limits for eggs and nymphs of both species (35°C) [18, 34, 35]. We predicted that intraspecific competition would be more intense (i.e., cause greater reductions in the fitness of individuals) under the optimal temperatures for each planthopper species and that this would counter the positive effects of optimal and elevated temperatures on the planthoppers. We also predicted that BPH would cause the least damage to temperate and tropical rice at optimal temperatures for the growth of each variety. However, because WBPH has slower nymph growth and development at higher temperatures (≥ 30°C), we expected this species to cause the greatest damage to plants in our experiments at 25°C. We discuss our results in light of the natural regulation of rice planthopper populations under future, warmer climates.

## Materials and methods

### Herbivores

We used BPH and WBPH from colonies maintained at the International Rice Research Institute (IRRI) in the Philippines. The colonies were initiated in 2009 with > 500 wild-caught individuals of each species collected from Laguna Province (Philippines: 14°10′N, 121°13′E). The planthoppers were reared continuously on the susceptible variety TN1 (≥ 30 days after sowing [DAS]) in wire mesh cages (91.5 × 56.5 × 56.5 cm; H × L × W). The colonies were kept under greenhouse conditions (26–37°C, 12:12 day:night) with feeding plants replaced every 3–5 days.

### Plant materials

We used two rice varieties in our experiments. IR22 is an *indica* rice variety that is susceptible to BPH and WBPH populations from South and Southeast Asia (moderately susceptible to populations from Bangladesh and Indonesia: [36]). T65 is a *japonica* variety from Taiwan. The variety is highly susceptible to BPH and WBPH from South and Southeast Asia [36]. Seeds of the two varieties were acquired through the IRRI Germplasm Collection. Seeds were germinated in climate chambers and planted to soil-filled pots (#0 pots = 7 × 11 cm: H × D) in the chambers for a bioassay to measure relative growth rates. Otherwise, the seeds were germinated in a greenhouse and planted at 5–6 days after sowing (DAS) to #0 pots filled with paddy soil. Before the competition experiments, the plants were placed under acetate cages (45×5cm: H×R). Each cage had a mesh side window and mesh top. The pots and developing plants were placed in climate chambers at the same temperatures as those used in the final competition bioassays. All infestations (see below) were to plants of 20 DAS. Excess, non-infested plants were used as controls to assess changes in biomass after planthopper attacks.

### Climate chamber experiments

Bioassays were conducted in environmental chambers with the Conviron CMP6050 Control System (Conviron, Winnipeg, Canada). Three temperature treatments, 25, 30 and 35°C were rotated between four separate chambers–changing the temperature settings after each experimental run. Relative humidity was maintained at 80% throughout the experiments. Each replicate (i.e., run) included between 1 and 6 subsamples (i.e., rearing cages–see below) per variety, and per intra- and interspecific planthopper density. Subsamples were randomized within chambers. The experiments were conducted as follows:

#### Temperature dependent plant growth rates

Seed of IR22 and T65 were germinated under constant temperatures of 25°C or 30°C. The germinated seed were transplanted to #0 pots and placed in climate chambers set at the same temperatures as for germination. Approximately 30 plants each of IR22 and T65 were placed in each chamber at arbitrary, but interspersed positions. Plants of each variety and from each chamber were randomly selected and destructively sampled at each of 22 time points (ranging from 2 to 40 DAS). The plants were cut above the soil, placed in individual paper bags and were dried in a forced draught oven at 60°C for 7 days. The experiment was replicated over two periods of about 50 days, i.e., a total of four runs each for plants (IR22 and T65) at 25 and 30°C (N = 4).

#### Intraspecific competition during oviposition

Plants were infested with recently emerged (≤ 3 days), gravid females of either BPH or WBPH at one of seven densities (1, 2, 4, 6, 8, 10 and 12 females) with densities randomly assigned to plants of each variety in chambers set at 25, 30 or 35°C. Densities were selected based on the numbers of adult females observed on rice plants of 15–30 DAS at sites in the Philippines. Although average numbers on young seedlings are typically between 1 and 4 per plant for adult females of both species, higher densities (i.e., 20 per plant) can occur under intensive rice production, particularly for WBPH. Higher densities of adult BPH (>30 per plant) are typical at later crop stages, particularly where fields have been treated with resurgence insecticides [19, 37–39]. There were between 3 and 6 sub-replicates per planthopper species, variety, density and temperature, depending on the availability of healthy plants and planthoppers at the beginning of each experimental run. Experiment were replicated five times (N = 5, as indicated above). For each run, plants were all infested on the same day. The adults were allowed to feed and oviposit for 7 days after which, the plants were cut above the soil and were dissected under a binocular microscope (× 10 magnification) to count egg clusters and eggs. Plants were then dried in a forced draft oven at 60°C for 7 days before weighing.

#### Intraspecific competition between planthopper nymphs

Neonate planthoppers were collected from the BPH and WBPH colonies and carefully placed into the cages using a handmade pooter. Plants in chambers at 25, 30 and 35°C were infested with nymphs of either BPH or WBPH at five densities (5, 10, 15, 20 and 25 nymphs) with densities randomly assigned to plants of each variety. Densities were selected based on records of field densities of both species on seedlings of 15 to 30 DAS in the Philippines. Densities of 7 to > 30 WBPH nymphs per plant have been observed on unsprayed rice seedlings during the wet season; whereas densities of BPH nymphs are typically lower on rice seedlings (i.e., < 3 per plant) in the Philippines, densities of > 30 per seedling have been recorded in fields treated with resurgence insecticides ([19, 37–39]). There were between 1 and 6 sub-replicates per planthopper species, variety, density and temperature, depending on the availability of healthy plants and planthopper numbers at the beginning of each experimental run. The experiment was replicated five times (N = 5, as indicated above). During each run, plants were all infested on the same day.

Nymphs were allowed to feed and develop for 15 days. Previous experiments using the same planthopper colonies and rice varieties have indicated that nymphs of both species will develop to adults in ≤ 10 days at temperatures of 25–30°C; nymphs will not develop to adults at 35°C [18]. After 15 days, the survivors were collected by tapping the cages over funnels into glass vials. The planthoppers were placed in cold storage before examination under a binocular microscope (×10 magnification) to determine development stages. After examination, the samples were dried at 60°C in a forced draft oven for 7 days before being weighed together (i.e., all planthoppers from a single plant) using a precision balance (Sartorius - 0.001g). The plants were cut above the soil and were also dried in a forced draft oven at 60°C for 7 days before weighing.

### Data analyses

Plant growth rates were analyzed using a repeated measures general linear model (GLM) with variety, temperature and their interaction as the main factors. The effects of temperature, variety, infestation density and their interactions on the number of eggs laid by gravid females and on the biomass, survival and development of nymphs were examined separately for each planthopper species using univariate GLM. Experimental run and plant weight were initially included in the models as random and covariate factors, respectively. Because of low survival (usually < 60%), slow development (nymphs rarely reached the 3rd instar) and low weight gain (≈ 5% of the weights achieved at lower temperatures) of nymphs at 35°C, we excluded this temperature from analyses of nymphs. The loss of biomass from rice seedlings due to planthopper feeding was estimated as the difference between the biomass of control (non-infested) plants and plants exposed to planthoppers. Weight losses per female and per unit weight of nymph were also estimated. We assessed the effects of exposure to adults and nymphs at different densities under the three test temperatures on plant biomass and on estimated weight losses using univariate GLMs. When grown under controlled conditions, the coefficients of variation in plant biomass ranged from 0.02 to 0.16, N = 5). Because of slow growth of nymphs at 35°C, variability in plant biomass often exceeded estimated yield losses from planthoppers; therefore, we excluded plant biomass losses per nymph weight at 35°C from the analyses. Following all parametric analyses, residuals were plotted and found to be normal and homogeneous. Density effects on egg-laying, nymph development, nymph weights and damage estimates were also described using best fit models. We used all data (from each replicate) to develop the models, but graphs are presented using means and standard errors. Models were assessed for normality and homogeneity of residuals.

## Results

### Temperature dependent plant growth rates

**Fig 1:**
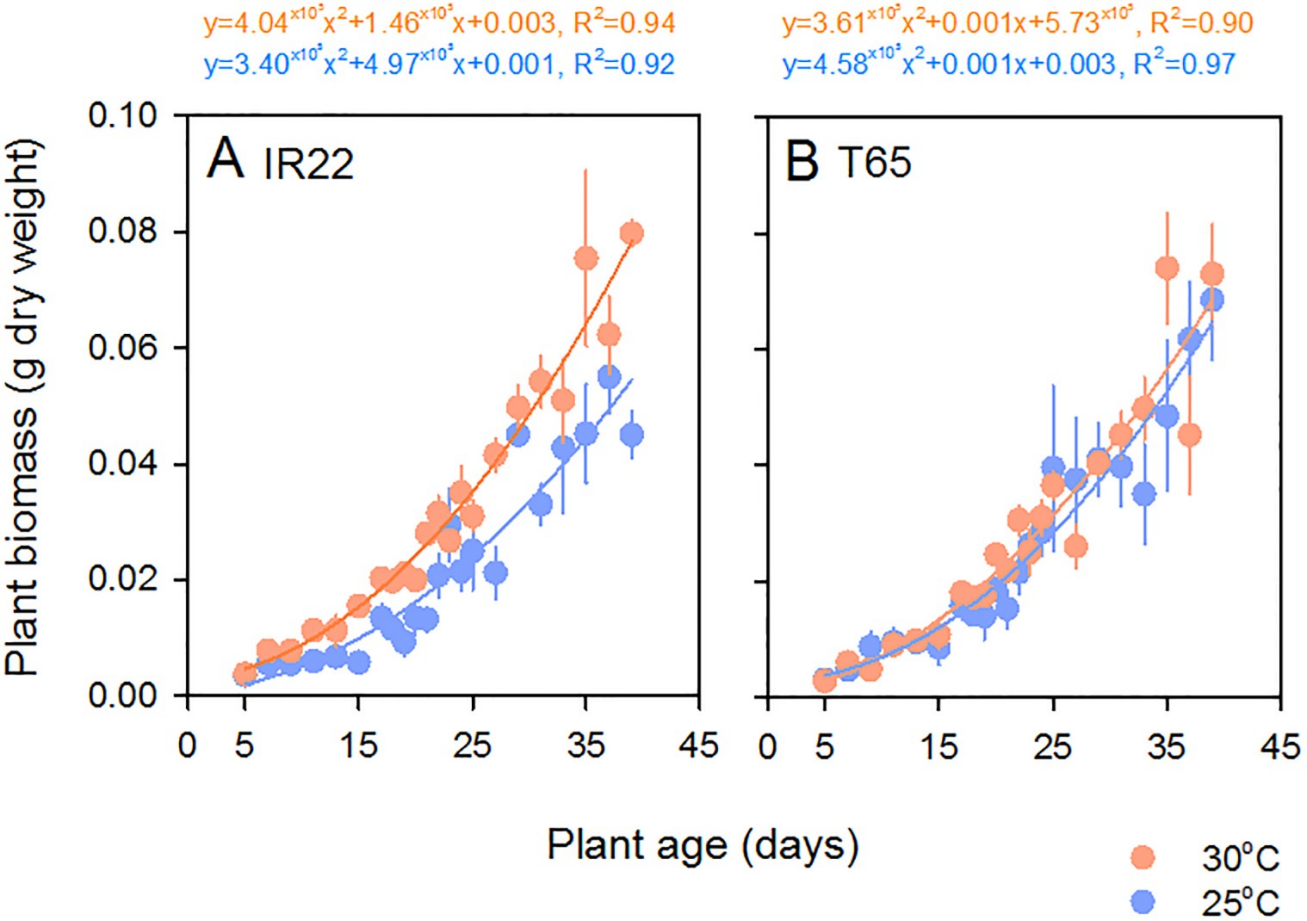
Growth of rice plants at 25°C and 30°C in constant temperature chambers. Points indicate (A) the biomass of IR22 plants at 25°C (blue symbols) and 30°C (orange symbols); and (B) the biomass of T65 plants at 25°C (blue symbols) and 30°C (orange symbols). Bars indicate standard errors (N = 4). Equations are best-fit models for each curve (corresponding colours) with associated R^2^ values.

Plants gained biomass over the course of 40 days in the climate chambers (Time: F_21,257_ = 58.447, P < 0.001) (Fig 1). There was no effect of variety on biomass over the course of the experiment (F_1,12_ = 0.006, P = 0.941). Plants grew larger at 30°C (Temperature: F_1,12_ = 16.999, P = 0.001); however, the effect was consistently greater in IR22 plants (i.e., across all time points: Fig 1A), resulting in a significant [temperature*variety] interaction (F_1,12_ = 7.042, P = 0.021). Growth was best described by quadratic models for each variety and temperature (Fig 1).

### Intraspecific competition during oviposition in BPH

**Fig 2:**
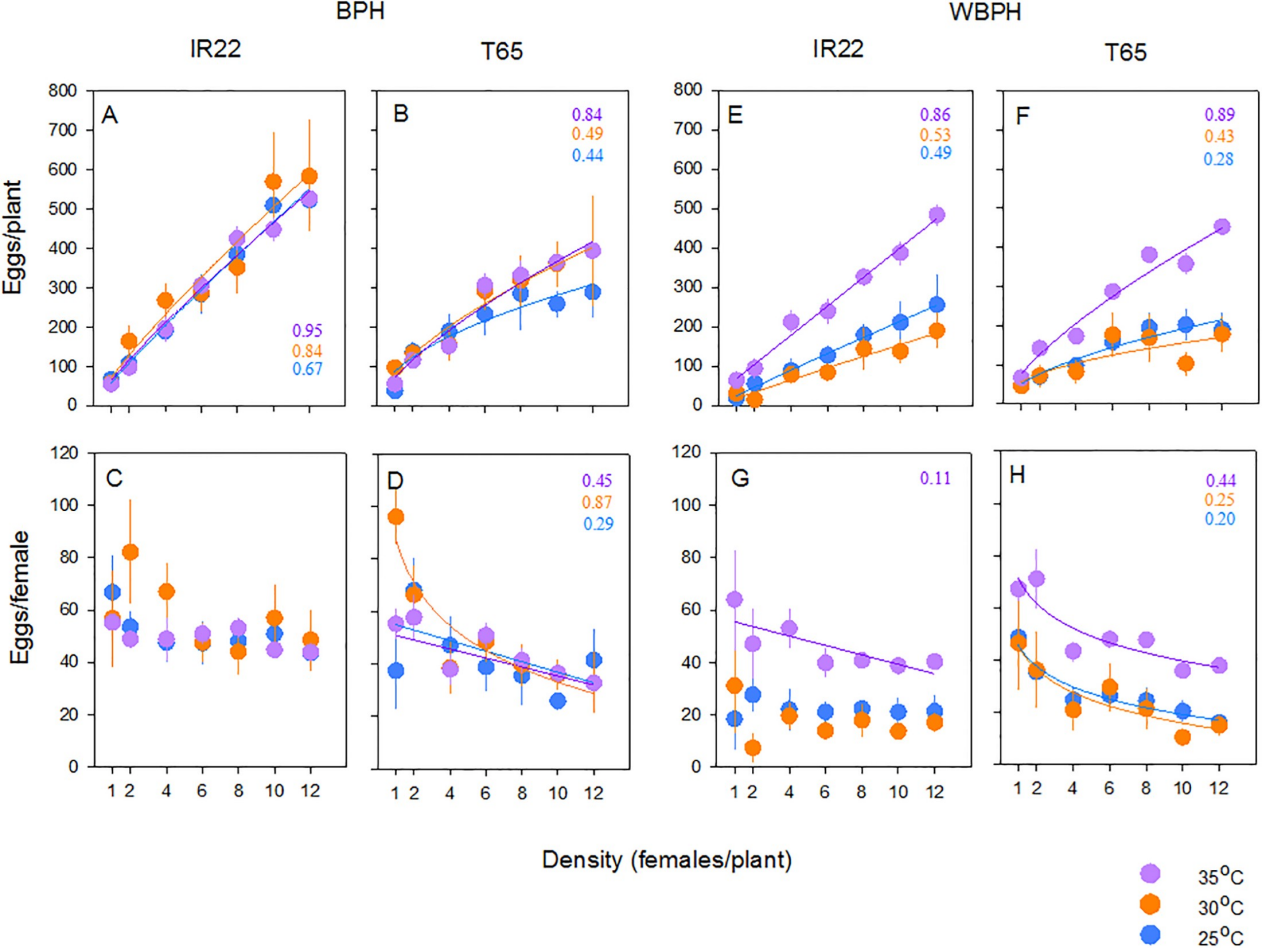
Relations between adult BPH (A,B) and WBPH (E,F) densities and the total number of eggs laid on IR22 (A,E) and T65 (B,F). The numbers of eggs laid per female BPH (C-D) and WBPH (G,H) on IR22 (C,G) and T65 (D,H) are also indicated. Bioassays were conducted in environmental chambers set at 25°C (blue symbols), 30°C (orange symbols) and 35°C (purple symbols). Means are indicated with standard errors (N = 5). Numbers are R^2^ values for best fit curves (as indicated; see S1 and S2 Tables).

BPH laid more eggs on IR22 plants than on T65 plants. More eggs were laid as the number of planthoppers per plant increased (Fig 2A and 2B; Table 1). For most temperature and variety combinations, these relations were best described by power functions (S1 Table), indicating that the planthoppers experienced intraspecific competition at higher densities. However, the effect was weak and was apparent as fewer eggs per female under increasing female densities only on T65 (at 25, 30 and 35°C: S2 Table; Fig 2C and 2D). Temperature did not affect oviposition (Fig 2A and 2B; Table 1).

**Table 1:** Results of univariate GLMs for the effects of BPH and WBPH female densities on oviposition.

Source of variation	DF	BPH^a^		WBPH^a^	
		Total eggs	Eggs per female	Total eggs	Eggs per female
Temperature (T)	2	0.759	0.820	26.541***	30.004***
Variety (V)	1	9.690***	9.697***	7.517**	2.331
Density (D)	6	78.637***	4.765***	34.060***	4.128***
Plant weight^b^	1	2.879	2.553	13.703***	25.939***
T×V	2	0.783	0.830	0.055	0.966
T×D	12	0.885	0.881	0.646	0.450
V×D	6	1.391	1.291	1.889	1.180
T×V×D	12	1.404	1.427	0.933	0.587
Error	167				

F-values are presented for the total numbers of eggs laid per plant and the numbers of eggs laid per individual female (see Fig 2).

^a^: *** = P < 0.005, ** = P < 0.01.

^b^: Plant weight was used as a covariate in the model.

### Intraspecific competition during oviposition in WBPH

WBPH laid more eggs on IR22 than on T65 and at higher planthopper densities (Fig 2E and 2F; Table 1). The relations between egg laying and density were generally best described by power functions (except for planthoppers on IR22 at 35°C: S1 Table), indicating that in most cases, planthoppers at high densities experienced intraspecific competition. The effects were greatest on T65 with females laying fewer eggs as densities increased at each of the three temperatures (S2 Table; Fig 2G and 2H). Temperature affected egg laying (Table 1): more eggs were laid by WBPH at 35°C at each planthopper density and on both varieties. There was no significant difference in egg-laying by WBPH at 25°C and 30°C (Fig 2E and 2F).

### Intraspecific competition between BPH nymphs

**Fig 3:**
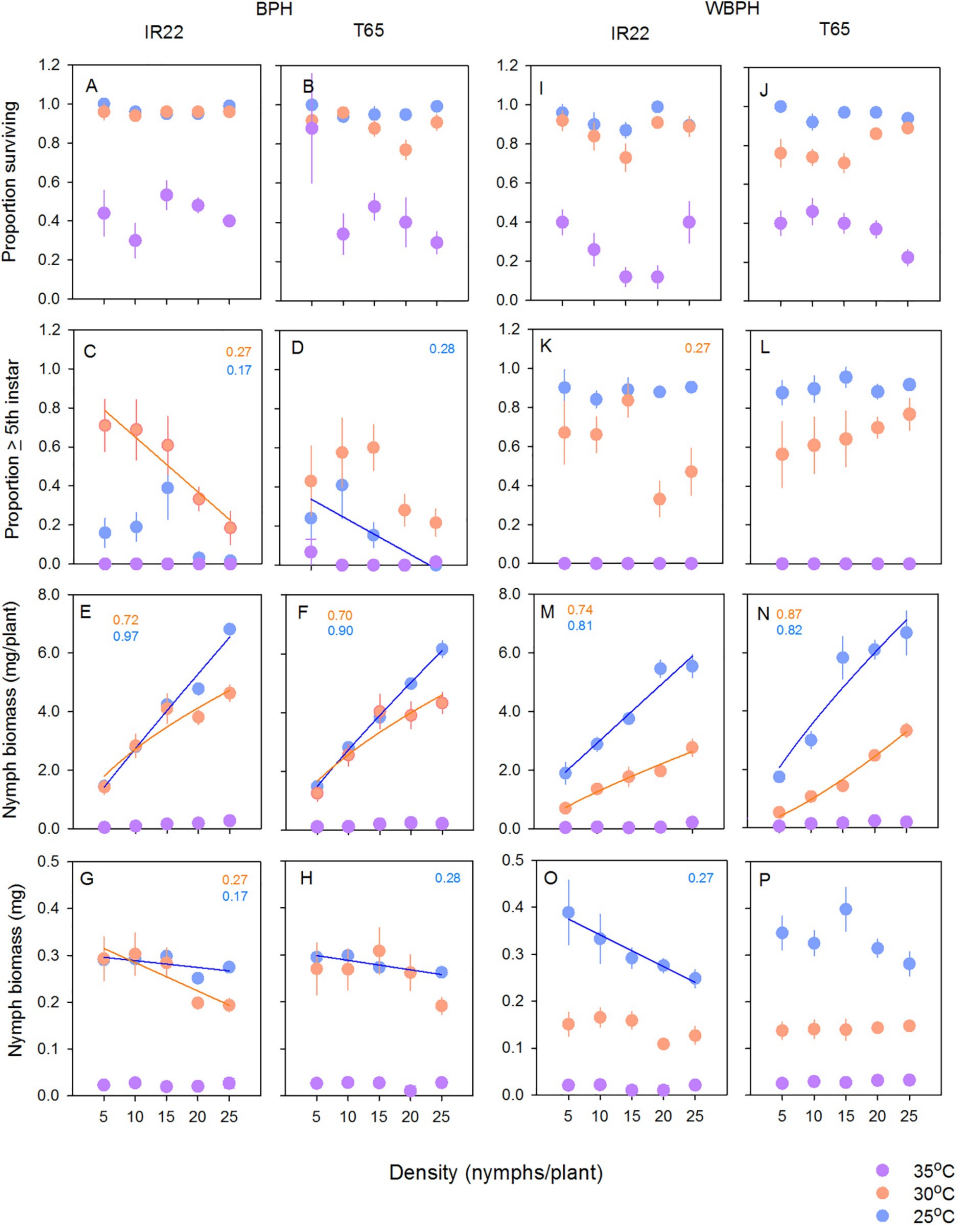
Relations between BPH (A-H) and WBPH (I-P) nymph densities and nymph survival (A,B,I,J), nymph development (C,D,K,L), the total weight of nymphs (E,F,M,N) and the average weights of individual nymphs (G,H,O,P). Nymphs were reared on IR22 (A,C,E,G,I,K,M,O) and T65 (B,D,F,H,J,L,N,P). Bioassays were conducted in environmental chambers set at 25°C (blue symbols), 30°C (orange symbols) and 35°C (purple symbols). Means are indicated with standard errors (N = 5). Numbers are R^2^ values for best fit models (as indicated; see S3–S5 Tables).

BPH nymphs had greater survival on IR22 than on T65, and survived better at 25°C compared to 30°C and at lower densities (Table 2; Fig 3A and 3B). An average of 46% of nymphs had developed beyond the fifth instar at 30°C, but only 15% at 25°C; however, the proportion of nymphs developing beyond the 5^th^ instar declined at both temperatures under conditions of increased crowding (Table 2, S3 Table; Fig 3C and 3D). At higher densities, nymphs gained greater biomass when feeding at 25°C than at 30°C, indicated by a significant [temperature*density] interaction (Table 2; Fig 3E and 3F). Slower development at 25°C compared to 30°C, but greater weight gains at the lower temperature under high nymph densities, produced comparatively heavier nymphs of each instar at 25°C (Fig 3G and 3H). Power models best-fit the nymph weight data in each case (variety and temperature) indicating that the nymphs experienced competition at the higher densities (S4 Table; Fig 3E–3H). These trends were also reflected in the data for individual nymph weights (Table 2; Fig 3G and 3H), but the relations were significant in only a few cases (S5 Table).

**Table 2:** F-values from univariate GLMs for nymph survival and development in environmental chambers at two temperatures (see Fig 3).

Source of variation	DF	F-values^a^							
		Nymph survival		Nymph development beyond 5^th^ instar		Nymph biomass per plant		Individual dry weight	
Planthopper		BPH	WBPH	BPH	WBPH	BPH	WBPH	BPH	WBPH
Temperature (T)	1	6.085*	27.098***	38.523***	43.525***	16.606***	454.56***	4.212*	334.395***
Variety (V)	1	4.027*	0.524	2.072	0.140	1.471	0.132	0.0431	0.501
Density (D)	4	3.253*	3.756**	8.498***	1.830	115.771***	127.646***	4.121***	1.620
Plant weight	1	3.610	0.010	4.270*	6.947**	0.390	16.558***	1.340	17.214***
T*V	1	0.403	6.702**	1.810	0.490	0.133	9.274***	0.030	10.486***
T*D	4	0.792	1.773	0.921	0.100	3.066*	3.702**	1.580	3.435**
V*D	4	0.593	1.391	0.650	1.320	0.313	3.170*	0.620	2.480*
T*V*D	4	1.551	0.580	1.123	0.950	0.174	1.590	0.460	1.241
Error	79								

^a^: *** = P < 0.001, ** = P < 0.01, * = P < 0.05; data for survival and development were arcsine-transformed and weight data were log-transformed before analysis.

### Intraspecific competition between WBPH nymphs

Survival of WBPH nymphs was lower at 30°C than at 25°C. Nymphs survived better at higher densities and there was a significant [temperature*variety] interaction because survival on IR22 was higher than on T65 at 30°C but the opposite trend occurred at 25°C (Fig 3I and 3J; Table 2). WBPH nymphs developed faster (Fig 3K and 3L) and gained greater biomass (Fig 3L and 3M) at 25°C than at 30°C (Table 2). Nymph biomass was greater on T65 but only at higher densities producing a significant [variety*density] interaction, and only at 25°C producing a significant [variety*temperature] interaction. A significant [temperature*density] interaction was due to similar biomass at low densities under 25°C and at high densities under 30°C (Table 2). Similar trends were noted when nymph dry weights per individual were analysed (Table 2; Fig 3O and 3P). Intraspecific competition was greatest on IR22 at 25°C as indicated by a power relation between density and total nymph biomass, and a linear decline in nymph weight (S4 and S5 Tables, Fig 3M–3P). At 30°C, low weight gains meant that WBPH nymphs did not show signs of intraspecific competition (Table 2).

### Changes in plant biomass in response to BPH adults

**Fig 4:**
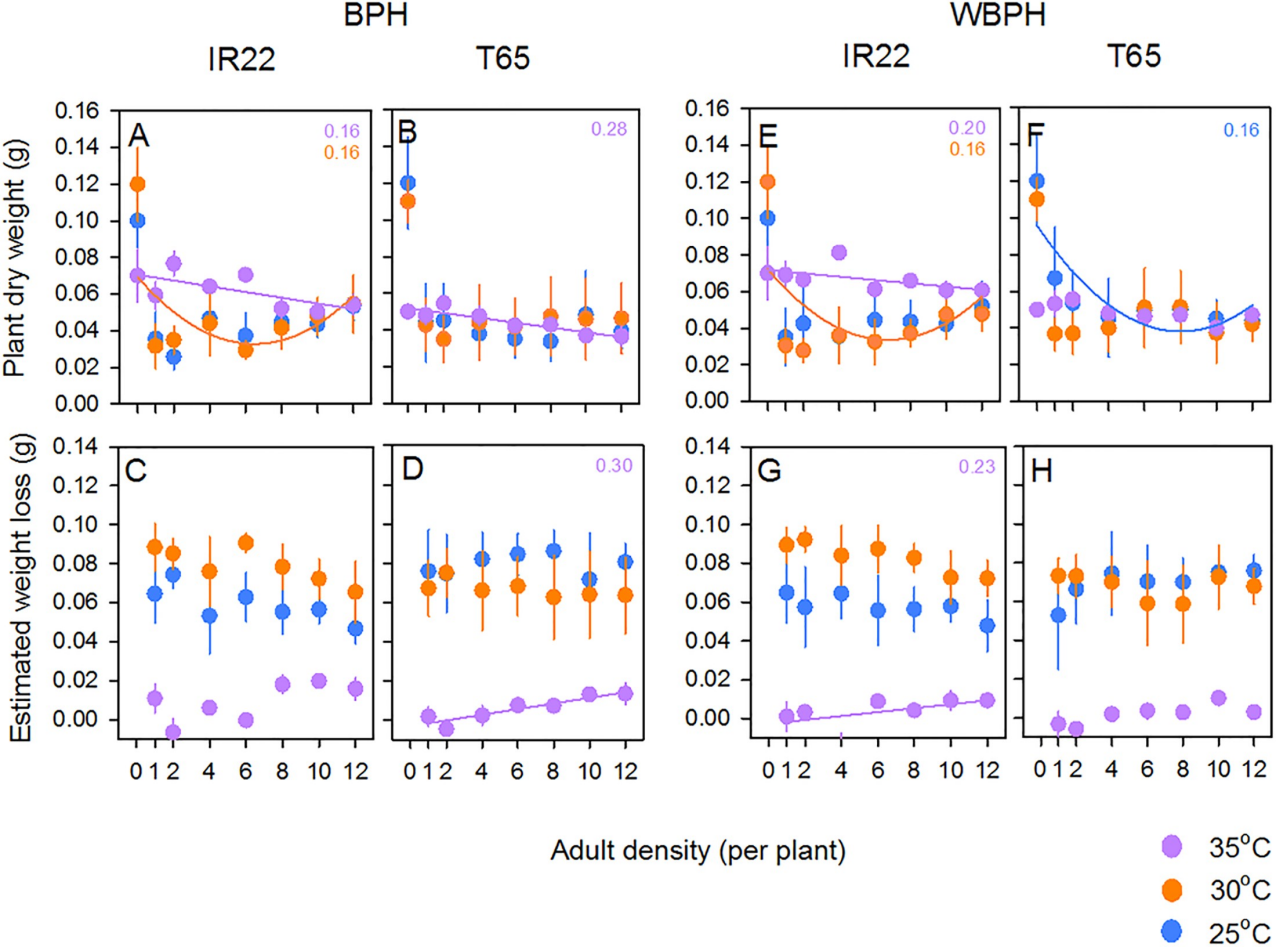
Effects of adult planthoppers on rice seedlings at three temperatures. The biomass of (A) IR22 seedlings and (B) T65 seedlings without exposure to planthoppers (density = 0) and after exposure to adult female BPH at densities ranging from 1 to 12 per plant are presented. Corresponding losses to the biomass of (C) IR22 and (D) T65 at different densities of adult BPH under the same conditions are also presented. Plants and planthoppers were maintained at three temperatures (25°C [blue symbols], 30°C [orange symbols] and 35°C [purple symbols]). The biomass of (C) IR22 and (B) T65 seedlings after exposure to adult WBPH females are presented with corresponding losses to (G) IR22 and (H) T65 due to WBPH adults. Standard errors are indicated (N = 5). Significant models and corresponding R^2^ values are included (colours as for symbols: see S6 and S7 Tables).

Feeding and egg laying by adult BPH reduced plant biomass (i.e., density = 0 compared to infested); however there was no significant difference between infested plant biomass under different densities of planthoppers (i.e., 1–12 based on Tukey tests) (Fig 4A and 4B; Table 3). The final biomass of plants at the end of the experiments was not affected by temperature or variety (Table 3). There was a significant [temperature*variety] interaction because of a lower biomass of T65 plants compared to IR22 at 35°C, and a significant [temperature*density] interaction because of the higher biomass of uninfested plants (planthopper density = 0) at 25 and 30°C compared to 35°C, but similar plant biomass of infested plants at all temperature (Fig 4A and 4B; Table 3). Adult BPH caused similar biomass losses at 25 and 30°C, but losses due to the planthoppers were negligible at 35°C irrespective of variety or adult density (Fig 4C and 4D; Table 3). There was a significant [temperature*variety] interaction for biomass loss because of greater losses to IR22 plants at 30°C, but greater losses to T65 plants at 25°C (Fig 4C and 4D). Information on losses per BPH adult is presented in S1 Fig.

**Table 3:** Results from univariate GLMs for plant biomass and estimated biomass loss at the end of experiments (see Fig 3).

Source of variation	Adult females^a^					Nymphs^a^				
	DF^b^	BPH		WBPH		DF^b^	BPH		WBPH	
		Plant biomass	Weight loss	Plant biomass	Weight loss		Plant biomass	Weight loss	Plant biomass	Weight loss
Temperature (T)	1	0.339	121.453***	1.565	141.853***	1	415.856***	10.805***	439.176***	11.923***
Variety (V)	2	2.226	0.219	0.315	0.300	2	11.511***	0.382	39.656***	3.280
Density (D)	7 (6)	8.301***	0.161	7.270***	0.061	5 (4)	6.191***	0.272	11.525***	0.308
T*V	2	2.941*	6.572***	5.223**	4.282**	2	1.73	2.373	4.889**	4.453*
T*D	14 (12)	1.885*	0.621	1.931*	0.376	10 (8)	2.221*	1.724	1.295	0.516
V*D	7 (6)	0.228	0.333	0.372	0.378	5 (4)	0.954	0.918	0.500	0.255
T*V*D	14 (12)	0.289	0.288	0.325	0.317	10 (8)	1.092	0.970	0.639	0.315
Error	180 (168)					144 (120)				

Numbers are F-values from analyses of plant biomass after feeding and oviposition by adult BPH and WBPH and of estimated biomass loss. F-values for analyses of plant biomass after nymph feeding by BPH and WBPH and corresponding estimates of biomass loss are also included.

^a^: *** = P < 0.001, ** = P < 0.01, * = P < 0.05.

^b^: Degrees of freedom are indicated for eight densities (i.e., 0 to 12 adults per plant) and six densities (i.e., 0 to 25 nymphs per plant) with corresponding degrees of freedom for planthopper related biomass loss for seven densities (i.e., 1 to 12 adults per plant) and five densities (i.e., 5 to 25) indicated in parentheses.

### Changes in plant biomass in response to WBPH adults

Feeding and egg laying by adult WBPH caused a reduction in plant biomass, but biomass did not differ across the range of planthopper densities used in the experiments (i.e., 1–12 adults per plant), and was not affected by temperature or variety (Fig 4E and 4F; Table 3). There was a significant [temperature*variety] interaction because of a lower biomass of T65 plants compared to IR22 at 35°C, and a significant [temperature*density] interaction because of higher biomass of uninfested plants (planthopper density = 0) at 25 and 30°C compared to 35°C, but similar plant biomass of infested plants at all temperature (Fig 4E and 4F; Table 3). Biomass losses were similar at 25 and 30°C, but negligible at 35°C irrespective of variety or adult density (Fig 4G and 4H). Greater biomass losses to IR22 plants at 30°C compared to 25°C, but similar losses to T65 plants at both temperatures produced a significant [temperature*variety] interaction (Fig 4G and 4H; Table 3). Information on biomass losses per adult is presented in S1 Fig.

### Changes in plant biomass in response to BPH nymphs

**Fig 5:**
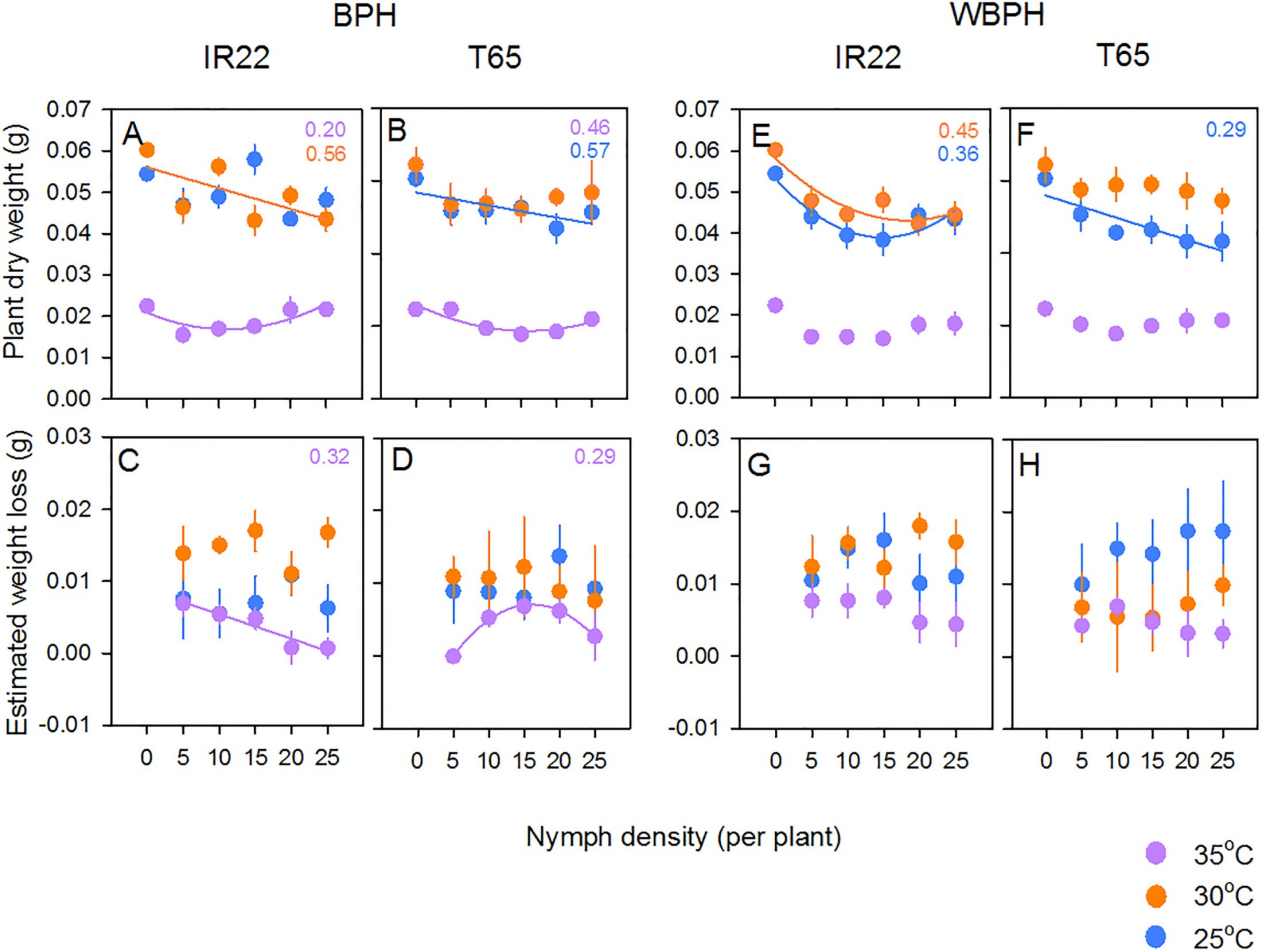
Effects of planthopper nymphs on rice seedlings at three temperatures. The biomass of (A) IR22 seedlings and (B) T65 seedlings without exposure to planthoppers (density = 0) and after exposure to BPH nymphs at densities ranging from 5 to 25 per plant are presented. Plants and planthoppers were maintained at three temperatures (25°C [blue symbols], 30°C [orange symbols] and 35°C [purple symbols]). Corresponding losses to the biomass of (C) IR22 and (D) T65 at different BPH nymph densities under the same conditions are also presented. The biomass of (E) IR22 and (F) T65 seedlings after exposure to WBPH nymphs are presented with corresponding losses to (G) IR22 and (H) T65 due to WBPH nymphs. Standard errors are indicated (N = 5). Significant models and corresponding R^2^ values are included (colours as for symbols: see S8 and S9 Tables).

BPH nymphs reduced plant biomass; however, biomass was not different between plants infested with different densities of nymphs (i.e., 5–25, based on Tukey tests) (Fig 5A and 5B; Table 3). At the end of the experiment, IR22 plants had lower biomass than T65 plants and plants were smaller at 35°C (Table 3). There was a significant [temperature*density] interaction because of similar plant biomass at 25 and 30°C at 5 nymphs per plant, but not at higher nymph densities (Fig 5A and 5B). Reductions in plants biomass due to BPH nymphs were greater at 30°C than at 25 or 35°C (Fig 5C and 5D; Table 3). Information on losses per unit biomass of nymphs is presented in S2 Fig.

### Changes in plant biomass in response to WBPH nymphs

WBPH nymphs reduced plant biomass; however, infested plant biomass was not different between plants with different densities of nymphs (i.e., 5–25, based on Tukey tests) (Fig 5E and 5F; Table 3). At the end of the experiment, IR22 plants had lower biomass than T65 plants. Plant biomass was lowest at 35°C, and lower at 25°C than at 30°C (Fig 5E and 5F; Table 3). There was a significant [temperature*variety] interaction because of lower final biomass of T65 plants at 25°C compared to 30°C, but similar biomass of IR22 plants at both temperatures under the same conditions of insect feeding (Fig 5E and 5F; Table 3). Biomass loss was higher at 25 and 30°C than at 35°C (Fig 5G and 5H; Table 3). There was a significant [temperature*variety] interaction because of greater losses to T65 biomass from WBPH nymphs feeding at 25°C than at 35°C, but similar biomass losses to IR22 plants across temperatures (Fig 5H; Table 3). Information on losses per unit biomass of nymphs is presented in S2 Fig.

## Discussion

BPH nymphs had more rapid development at 30°C and attained relatively high weights (at low densities) and high survival at 25 and 30°C. WBPH nymphs had higher survival, more rapid development and attained a greater biomass at 25°C compared to 30°C. Nymphs of both species failed to develop to adults at 35°C possibly due to adverse effects of the high temperature on yeast-like endosymbionts, which are essential for nymph nutrition [18, 40–42]; but also due to the comparatively slow growth of rice plants at 35°C. Adult BPH produced more eggs at 30°C than at 25 or 35°C. Contrary to expectations, WBPH females produced more eggs at 35°C than at other temperatures during our experiments. However, a previous study has shown that WBPH adult longevity is severely reduced at 35°C and, consequently, life-time fecundities are generally lower at 35°C than at 25 or 30°C (egg-laying was similar at 25 and 30°C) [18]. We suggest that the high rate of oviposition in WBPH at 35°C was a stress response. Planthopper eggs deposited at such high temperatures have reduced (by ≈ 60%) hatchability compared to eggs deposited at 25–30°C [43–45].

Variety had little effect on planthopper life histories, except that BPH laid more eggs on IR22 than on T65 at all temperatures. On T65, intraspecific competition reduced egg-laying by > 65% (> 50% per individual) in both species at high densities (12 per plant). Greater competition between ovipositing BPH and WBPH on T65 (indicated by significant declines in fecundity at higher densities) compared to IR22 was probably due to the anatomy of the rice plants and the availability of space at the base of the stems for oviposition [19, 46]. Our results suggest that space was more limited on T65 than on IR22. Temperature had no significant effect on competition between ovipositing females of either species. In contrast to adults, intraspecific competition between nymphs was generally most intense at the optimal temperatures for each planthopper species. For example, at high densities (20–25 per plant) the biomass of WBPH nymphs declined sharply at 25°C. At suboptimal temperatures (35°C for BPH and ≥ 30°C for WBPH) nymphs were not significantly affected by conspecific densities. The effects of suboptimal temperatures on intraspecific competition in planthoppers are similar to the effects of resistant rice on intraspecific planthopper interactions [20, 23] suggesting that sub-optimal temperatures reduce the availability or quality of resources for the planthoppers and/or reduce resource acquisition.

In a previous study, increasing temperatures from 25°C to 30°C caused a reduction in WBPH nymph survival by ≈ 12% and in biomass by ≈ 25%, but oviposition and nymph development did not change significantly [18]. Our results suggest that at ≥ 30°C, intraspecific competition will have little further impact on the fitness of WBPH nymphs. However, at about the optimal temperature, moderate to high densities (15–25 per plant) reduced WBPH nymph biomass by up to 30%. Such densities of WBPH nymphs are common in Asian rice fields, particularly during early crop stages [37]. Where fields are carefully managed to maintain natural enemies, WBPH densities will typically decline to very few individuals per plant at later crop stages [37]. Our results suggest that competition between nymphs at optimal temperatures contributes to WBPH population stability in rice and to the declines observed as the crop matures. Population regulation by natural enemies [37, 47, 48] and ontogenic changes in rice susceptibility [46] also contribute to the decline of WBPH populations during crop maturation. Our results indicate that current ambient temperatures in tropical Asia, that frequently surpass 30°C during extended periods [18, 41], will reduce the potential for WBPH to attain outbreak densities.

Increasing temperatures from 25 to 30°C increases BPH fecundity by ≈ 20% [18]. This is apparently due to increased feeding by gravid females at the higher temperature and the conversion of resources to eggs during discrete oviposition cycles [21, 41]. However, in contrast to fecundity, increasing temperatures from 25 to 30°C has been shown to reduce BPH nymph biomass by ≈ 35% without affecting nymph survival or development rates [18]. In our experiments, the biomass of BPH nymphs in chambers at 30°C, also declined by up to 35% compared to nymphs at 25°C, but only at high densities (20–25 per plant). This suggests that the apparently lower optimal temperature for BPH nymphs to gain biomass (i.e., 25 versus 30°C) may be an artefact due to the relatively high densities of nymphs used in previous studies (i.e., 10 nymphs per 15 DAS plant [18]). Nymphs at lower densities (i.e., 5–15 per 20 DAS plant) performed equally well at 25 and 30°C in our experiments. Such low densities are typical of BPH in well managed tropical rice fields [37]. BPH will occur in rice fields at the highest densities used in our experiments, but generally only during fertilizer or pesticide induced outbreaks where natural enemy numbers are reduced [38, 39]. Our results indicate that intraspecific competition will contribute to population stability during such outbreaks, and that the effect will be greater at higher temperatures.

Greater declines in BPH biomass at 30°C compared to 25°C under increasing nymph densities suggest that competition between nymphs at the higher temperature was not due to increased resource exploitation. For example, at 30°C, IR22 plants grew larger than at 25°C, but nymphs were smaller. The production of larger BPH nymphs at 25°C may be explained by the temperature-size rule [49, 50]. At 25°C, BPH nymphs may have gained greater biomass because of their slower development (that became further delayed under high densities) and consequent extended feeding. However, our results also suggest that weight advantages gained through the temperature-size rule may be partly explained by a relaxation of intraspecific competition–most probably because of a reduction in direct or plant-mediated interference between individuals during feeding at the lower temperature (compared to 30°C). For example, several studies have indicated that the frequency of aggressive encounters between planthoppers increases at higher temperatures [51, 52]. Furthermore, plant-mediated interference competition between WBPH and BPH has been suggested to neutralize the positive effects of elevated temperature (i.e., 30°C) on BPH population growth on IR22 and T65, despite poor growth and development of WBPH at the same high temperature; the effect was less pronounced at a lower temperature (25°C) [14]. The decoupling of resource use and both intra- (this study) and interspecific [14] competition suggests that plant defences or other interference mechanisms induced by planthoppers feeding on IR22 and T65 may be more effective at the high temperature.

Plant biomass was severely reduced by exposure to adult female planthoppers. For example, by the end of our experiments, plants infested at 25 and 30°C had similar or lower biomass to plants at 35°C–where growth had been curtailed by the high temperature. In the oviposition experiments, we estimate that plants at 35°C lost relatively little biomass, despite often high numbers of eggs deposited. This indicates that biomass was lost mainly due to adult feeding and not oviposition. We predicted that IR22 would be less affected by planthoppers at 30°C than at 25°C because of faster growth rates at the higher temperature, and that the effect would be greater for plants infested with WBPH compared to those infested with BPH, because the higher temperature had clearly adverse effects on WBPH, particularly during the nymph stages. However, contrary to predictions, we estimated greater biomass losses to IR22 plants when exposed to adults of either species at the higher temperature. Although T65 plants had similar growth rates at 25 and 30°C in our initial growth rate experiment, control plants were larger at 25°C compared to 30°C in our oviposition experiments. These T65 plants tended to lose more biomass when infested by adults of both species at 25°C, despite greater egg-laying and presumably more feeding by adult BPH at 30°C.

We estimated that BPH nymphs caused greater losses to plant biomass at 30°C compared to 25°C—particularly in IR22—despite often lower BPH biomass at 30°C. Meanwhile, WBPH nymphs caused a greater reduction in T65 biomass at 25°C. However, despite the considerably lower biomass of WBPH nymphs at 30°C, estimated reductions in the biomass of IR22 plants were similar (at 5–15 nymphs per plant) or higher (at 20–25 nymphs per plant) at 30°C compared to 25°C. Therefore, our results with adults and nymphs of both species consistently indicated that rice plants were more severely affected by planthoppers at the optimal temperatures for the varieties and not for the insects. These results contrast with those form a range of systems that indicate greater tolerance to herbivores under optimal conditions (i.e., nitrogen, light, etc.) for plant growth [53, 54]. Therefore, in our experiments, estimated herbivore-related losses in plant biomass were decoupled from planthopper resource use (i.e., eggs laid or biomass gained) at different temperatures. One possible explanation for these trends is that the planthoppers in our experiments may have induced plant responses that were more costly (including defence costs and/or maintenance costs) to the plants at the optimal temperatures for each variety. It is also possible that rapid growth under optimal temperatures may have depleted soil nutrients to a greater extent than under sub-optimal temperatures, with planthopper feeding adding further, and proportionally greater, nutrient stresses to plants at the optimal temperatures for plant growth. Because we conducted our experiments using plants in small pots without added fertilizer, we suggest that our results represent situations where plants may be nutrient limited and that our observations on biomass losses may not be applicable to heavily fertilized rice fields. Tolerance to planthopper feeding can be augmented by adding soil nutrients (i.e., nitrogen for WBPH [55] or potassium for BPH [56]) to rice crops. Further research on the interactions between soil nutrients, herbivory and ambient temperatures is required to determine best management practices for phloem-feeders under increasingly warmer temperatures.

## Supporting information

S1 Table Best fit models to describe the relation between planthopper densities and egg numbers on two rice varieties at constant temperatures of 25°C, 30°C and 35°C. (DOCX)

S2 Table Best fit models to describe the relation between planthopper densities and egg laid per female on two rice varieties at constant temperatures of 25°C, 30°C and 35°C. (DOCX)

S3 Table Best fit models to describe the relation between nymph densities and the proportion of nymphs developing beyond the fifth instar on two rice varieties at constant temperatures of 25°C and 30°C. (DOCX)

S4 Table Best fit models to describe the relation between nymph densities and total nymph biomass on two rice varieties at constant temperatures of 25°C, 30°C and 35°C. (DOCX)

S5 Table Best fit models to describe the relation between nymph densities and individual nymph weight on two rice varieties at constant temperatures of 25°C, 30°C and 35°C. (DOCX)

S6 Table Best fit models to describe the relation between adult densities and final dry weight on two rice varieties at constant temperatures of 25°C, 30°C and 35°C. (DOCX)

S7 Table Best fit models to describe the relation between adult densities and estimated plant weight loss for two rice varieties at constant temperatures of 25°C, 30°C and 35°C. (DOCX)

S8 Table Best fit models to describe the relation between nymph densities and final dry weight of two rice varieties at constant temperatures of 25°C, 30°C and 35°C. (DOCX)

S9 Table Best fit models to describe the relation between nymph densities and plant weight loss for two rice varieties at constant temperatures of 25°C, 30°C and 35°C. (DOCX)

S10 Table Data for plant biomass gain at 25 and 30°C. (DOCX)

S11 Table Data from oviposition experiments. (DOCX)

S12 Table Data from nymph experiments. (DOCX)

S1 Fig Seedling weight loss per adult planthopper during oviposition experiments. (DOCX)

S2 Fig Seedling weight loss per nymph weight during experiments. (DOCX)
