# Supplementary material for: Intraspecific competition counters the effects of elevated and optimal temperatures on phloem-feeding insects in tropical and temperate rice
Source: PLoS One. 2020 Oct 6;15(10):e0240130. doi: 10.1371/journal.pone.0240130 (PMC7538200; doi:10.1371/journal.pone.0240130)
Supplement: S1 Table — (DOCX) [file pone.0240130.s001.docx]

**Table S1. Best fit models to describe the relation between planthopper densities and egg numbers on two rice varieties at constant temperatures of 25°C, 30°C and 35°C**

| Species | Variety | Temperature (°C) | Model^a^ | Constant | B1 | R^2^ | F-value^b^ | P-value |
| --- | --- | --- | --- | --- | --- | --- | --- | --- |
| BPH | IR22 | 25 | Power | 0.329 | 0.925 | 0.972 | 788.866 | 0.0001 |
| BPH | IR22 | 30 | Power | 0.440 | 0.750 | 0.718 | 58.471 | 0.0001 |
| BPH | IR22 | 35 | Power | 0.006 | 1.145 | 0.611 | 36.095 | 0.0001 |
| BPH | T65 | 25 | Power | 0.359 | 0.879 | 0.904 | 486.815 | 0.0001 |
| BPH | T65 | 30 | Power | 0.318 | 0.847 | 0.669 | 46.547 | 0.0001 |
| BPH | T65 | 35 | Logarithmic | -0.084 | 0.104 | 0.261 | 8.130 | 0.009 |
| WBPH | IR22 | 25 | Linear | 0.940 | 0.198 | 0.809 | 97.700 | 0.0001 |
| WBPH | IR22 | 30 | Power | 0.181 | 0.823 | 0.744 | 66.807 | 0.0001 |
| WBPH | IR22 | 35 | Quadratic | -0.023 | 0.001 | 0.461 | 9.414 | 0.001 |
| WBPH | T65 | 25 | Power | 0.407 | 0.896 | 0.822 | 106.077 | 0.0001 |
| WBPH | T65 | 30 | Power | 0.071 | 1.164 | 0.873 | 157.789 | 0.0001 |
| WBPH | T65 | 35 | Power | 0.014 | 0.861 | 0.556 | 28.803 | 0.0001 |

a: Models are indicated in Figure 2 A,B,E,F

b: Model DF = 1,23 for linear, logarithmic and power models and 2,22 for quadratic model
