# Supplementary material for: Intraspecific competition counters the effects of elevated and optimal temperatures on phloem-feeding insects in tropical and temperate rice
Source: PLoS One. 2020 Oct 6;15(10):e0240130. doi: 10.1371/journal.pone.0240130 (PMC7538200; doi:10.1371/journal.pone.0240130)
Supplement: S2 Table — (DOCX) [file pone.0240130.s002.docx]

**Table S2. Best fit models to describe the relation between planthopper densities and egg laid per female on two rice varieties at constant temperatures of 25°C, 30°C and 35°C**

| Species | Variety | Temperature (°C) | Model^a^ | Constant | B1 | R^2^ | F-value^b^ | P-value |
| --- | --- | --- | --- | --- | --- | --- | --- | --- |
| BPH | IR22 | 25 | Power | 58.008 | -0.120 | 0.089 | 3.239 | 0.081 |
| BPH | IR22 | 30 | Linear | 69.552 | -1.944 | 0.061 | 2.154 | 0.152 |
| BPH | IR22 | 35 | Linear | 53.692 | -0.694 | 0.081 | 2.926 | 0.097 |
| BPH | T65 | 25 | Logarithmic | 52.61 | -1.722 | 0.873 | 11.389 | 0.002 |
| BPH | T65 | 30 | Linear | 80.578 | -5.004 | 0.452 | 27.241 | 0.0001 |
| BPH | T65 | 35 | Linear | 56.892 | -2.006 | 0.288 | 13.344 | 0.001 |
| WBPH | IR22 | 25 | Power | 8.155 | 0.392 | 0.083 | 2.976 | 0.094 |
| WBPH | IR22 | 30 | Power | 7.724 | 0.248 | 0.041 | 1.413 | 0.243 |
| WBPH | IR22 | 35 | Linear | 57.417 | -1.813 | 0.106 | 3.912 | 0.055 |
| WBPH | T65 | 25 | Logarithmic | 45.633 | -11.543 | 0.253 | 11.157 | 0.002 |
| WBPH | T65 | 30 | Logarithmic | 45.421 | -2.697 | 0.203 | 8.417 | 0.007 |
| WBPH | T65 | 35 | Logarithmic | 71.06 | -13.654 | 0.441 | 30.518 | 0.0001 |

a: Significant models are indicated in Figure 2 C,D,G,H

b: Model DF = 1,33 for each case
