# Supplementary material for: Intraspecific competition counters the effects of elevated and optimal temperatures on phloem-feeding insects in tropical and temperate rice
Source: PLoS One. 2020 Oct 6;15(10):e0240130. doi: 10.1371/journal.pone.0240130 (PMC7538200; doi:10.1371/journal.pone.0240130)
Supplement: S3 Table — (DOCX) [file pone.0240130.s003.docx]

**Table S3. Best fit models to describe the relation between nymph densities and the proportion of nymphs developing beyond the fifth instar on two rice varieties at constant temperatures of 25°C, and 30°C**

| Species | Variety | Temperature (°C)^a^ | Model^b^ | Constant | B1 | R^2^ | F-value^c^ | P-value |
| --- | --- | --- | --- | --- | --- | --- | --- | --- |
| BPH | IR22 | 25 | Linear | 0.293 | -0.009 | 0.083 | 2.083 | 0.162 |
| BPH | IR22 | 30 | Linear | 0.927 | -0.028 | 0.382 | 14.222 | 0.001 |
| BPH | T65 | 25 | Linear | 0.427 | -0.018 | 0.259 | 8.038 | 0.009 |
| BPH | T65 | 30 | Linear | 0.637 | -0.014 | 0.111 | 2.877 | 0.103 |
| WBPH | IR22 | 25 | Power | 0.816 | 0.025 | 0.007 | 0.157 | 0.696 |
| WBPH | IR22 | 30 | Linear | 0.815 | -0.015 | 0.125 | 3.286 | 0.083 |
| WBPH | T65 | 25 | Power | 0.820 | 0.036 | 0.024 | 0.564 | 0.460 |
| WBPH | T65 | 30 | Power | 0.053 | 0.861 | 0.132 | 3.503 | 0.074 |

a: No nymphs reached the 5^th^ instar at 35°C

b: Models are indicated in Figure 3 C,D

c: Model DF = 1,23 for each case
