# Supplementary material for: Intraspecific competition counters the effects of elevated and optimal temperatures on phloem-feeding insects in tropical and temperate rice
Source: PLoS One. 2020 Oct 6;15(10):e0240130. doi: 10.1371/journal.pone.0240130 (PMC7538200; doi:10.1371/journal.pone.0240130)
Supplement: S5 Table — (DOCX) [file pone.0240130.s005.docx]

**Table S5. Best fit models to describe the relation between nymph densities and individual nymph weight on two rice varieties at constant temperatures of 25°C, 30°C and 35°C**

| Species | Variety | Temperature (°C) | Model^a^ | Constant | B1 | R^2^ | F-value^b^ | P-value |
| --- | --- | --- | --- | --- | --- | --- | --- | --- |
| BPH | IR22 | 25 | Linear | 0.303 | -0.001 | 0.172 | 4.793 | 0.039 |
| BPH | IR22 | 30 | Linear | 0.345 | -0.006 | 0.271 | 8.555 | 0.008 |
| BPH | IR22 | 35 | Power | 0.024 | -0.038 | 0.003 | 0.068 | 0.796 |
| BPH | T65 | 25 | Linear | 0.309 | -0.002 | 0.278 | 8.856 | 0.007 |
| BPH | T65 | 30 | Linear | 0.310 | -0.003 | 0.062 | 1.532 | 0.228 |
| BPH | T65 | 35 | Power | 0.030 | -0.094 | 0.006 | 0.132 | 0.719 |
| WBPH | IR22 | 25 | Power | 0.546 | -0.238 | 0.268 | 8.414 | 0.008 |
| WBPH | IR22 | 30 | Linear | 0.173 | -0.002 | 0.111 | 2.878 | 0.103 |
| WBPH | IR22 | 35 | Power | 0.038 | -0.451 | 0.053 | 1.292 | 0.267 |
| WBPH | T65 | 25 | Linear | 0.377 | -0.003 | 0.072 | 1.783 | 0.195 |
| WBPH | T65 | 30 | Power | 0.120 | 0.056 | 0.02 | 0.463 | 0.503 |
| WBPH | T65 | 35 | Linear | 0.025 | 0.001 | 0.167 | 4.627 | 0.042 |

a: Significant models are indicated in Figure 7 G,H,O,P

b: Model DF = 1,23 for each case
