# Supplementary material for: Intraspecific competition counters the effects of elevated and optimal temperatures on phloem-feeding insects in tropical and temperate rice
Source: PLoS One. 2020 Oct 6;15(10):e0240130. doi: 10.1371/journal.pone.0240130 (PMC7538200; doi:10.1371/journal.pone.0240130)
Supplement: S6 Table — (DOCX) [file pone.0240130.s006.docx]

**Table S6. Best fit models to describe the relation between adult densities and final dry weight on two rice varieties at constant temperatures of 25°C, 30°C and 35°C**

| Species | Variety | Temperature (°C) | Model^a^ | Constant | B1 | R^2^ | F-value^b^ | P-value |
| --- | --- | --- | --- | --- | --- | --- | --- | --- |
| BPH | IR22 | 25 | Quadratic | 0.062 | -0.009 | 0.094 | 1.812 | 0.178 |
| BPH | IR22 | 30 | Quadratic | 0.072 | -0.013 | 0.157 | 3.266 | 0.050 |
| BPH | IR22 | 35 | Linear | 0.071 | -0.002 | 0.162 | 6.942 | 0.012 |
| BPH | T65 | 25 | Quadratic | 0.078 | -0.013 | 0.141 | 2.878 | 0.070 |
| BPH | T65 | 30 | Quadratic | 0.07 | -0.010 | 0.068 | 1.272 | 0.293 |
| BPH | T65 | 35 | Linear | 0.051 | -0.001 | 0.278 | 13.893 | 0.001 |
| WBPH | IR22 | 25 | Quadratic | 0.066 | -0.009 | 0.084 | 1.595 | 0.217 |
| WBPH | IR22 | 30 | Quadratic | 0.07 | -0.130 | 0.164 | 3.425 | 0.044 |
| WBPH | IR22 | 35 | Linear | 0.075 | -0.001 | 0.197 | 8.853 | 0.005 |
| WBPH | T65 | 25 | Quadratic | 0.09 | -0.012 | 0.156 | 3.234 | 0.051 |
| WBPH | T65 | 30 | Linear | 0.057 | -0.001 | 0.029 | 1.069 | 0.308 |
| WBPH | T65 | 35 | Linear | 0.047 | 0.001 | 0.001 | 0.048 | 0.827 |

a: Significant models are indicated in Figure 4A,B,E,F

b: Model DF = 1,36 for linear models and 2,35 for quadratic models
