# Supplementary material for: Intraspecific competition counters the effects of elevated and optimal temperatures on phloem-feeding insects in tropical and temperate rice
Source: PLoS One. 2020 Oct 6;15(10):e0240130. doi: 10.1371/journal.pone.0240130 (PMC7538200; doi:10.1371/journal.pone.0240130)
Supplement: S7 Table — (DOCX) [file pone.0240130.s007.docx]

**Table S7. Best fit models to describe the relation between adult densities and estimated plant weight loss for two rice varieties at constant temperatures of 25°C, 30°C and 35°C**

| Species | Variety | Temperature (°C) | Model^a^ | Constant | B1 | R^2^ | F-value^b^ | P-value |
| --- | --- | --- | --- | --- | --- | --- | --- | --- |
| BPH | IR22 | 25 | Linear | 0.072 | -0.002 | 0.086 | 3.115 | 0.087 |
| BPH | IR22 | 30 | Linear | 0.092 | -0.002 | 0.076 | 2.706 | 0.109 |
| BPH | IR22 | 35 | Linear | 0.024 | 0.001 | 0.001 | 0.015 | 0.901 |
| BPH | T65 | 25 | Logarithmic | 0.077 | 0.002 | 0.003 | 0.092 | 0.764 |
| BPH | T65 | 30 | Linear | 0.071 | -0.001 | 0.003 | 0.107 | 0.746 |
| BPH | T65 | 35 | Linear | (-)0.002 | 0.001 | 0.304 | 14.433 | 0.001 |
| WBPH | IR22 | 25 | Linear | 0.064 | -0.001 | 0.018 | 0.601 | 0.444 |
| WBPH | IR22 | 30 | Linear | 0.094 | -0.002 | 0.07 | 2.488 | 0.124 |
| WBPH | IR22 | 35 | Linear | (-)0.005 | 0.001 | 0.232 | 9.975 | 0.003 |
| WBPH | T65 | 25 | Linear | 0.061 | 0.001 | 0.021 | 0.702 | 0.408 |
| WBPH | T65 | 30 | Inverse | 0.066 | -0.002 | 0.006 | 0.192 | 0.664 |
| WBPH | T65 | 35 | Linear | 0.036 | -0.002 | 0.049 | 2.849 | 0.097 |

a: Significant models are indicated in Figure 4C,D,G,H

b: Model DF = 1,33 for all models
