# Supplementary material for: Intraspecific competition counters the effects of elevated and optimal temperatures on phloem-feeding insects in tropical and temperate rice
Source: PLoS One. 2020 Oct 6;15(10):e0240130. doi: 10.1371/journal.pone.0240130 (PMC7538200; doi:10.1371/journal.pone.0240130)
Supplement: S8 Table — (DOCX) [file pone.0240130.s008.docx]

**Table S8. Best fit models to describe the relation between nymph densities and final dry weight of two rice varieties at constant temperatures of 25°C, 30°C and 35°C**

| Species | Variety | Temperature (°C) | Model^a^ | Constant | B1 | R^2^ | F-value^b^ | P-value |
| --- | --- | --- | --- | --- | --- | --- | --- | --- |
| BPH | IR22 | 25 | Linear | 0.522 | -0.002 | 0.044 | 1.285 | 0.267 |
| BPH | IR22 | 30 | Linear | 0.561 | -0.005 | 0.264 | 10.039 | 0.004 |
| BPH | IR22 | 35 | Quadratic | 0.209 | -0.007 | 0.233 | 4.109 | 0.028 |
| BPH | T65 | 25 | Linear | 0.568 | -0.003 | 0.147 | 4.836 | 0.036 |
| BPH | T65 | 30 | Quadratic | 0.628 | -0.015 | 0.104 | 1.571 | 0.226 |
| BPH | T65 | 35 | Quadratic | 0.26 | -0.009 | 0.461 | 11.545 | 0.001 |
| WBPH | IR22 | 25 | Quadratic | 0.532 | -0.019 | 0.357 | 7.5 | 0.003 |
| WBPH | IR22 | 30 | Quadratic | 0.582 | -0.016 | 0.445 | 10.816 | <0.001 |
| WBPH | IR22 | 35 | Quadratic | 0.211 | 0.211 | 0.283 | 5.337 | 0.11 |
| WBPH | T65 | 25 | Linear | 0.56 | -0.006 | 0.289 | 11.39 | 0.002 |
| WBPH | T65 | 30 | Linear | 0.622 | -0.003 | 0.085 | 2.603 | 0.118 |
| WBPH | T65 | 35 | Quadratic | 0.239 | -0.007 | 0.187 | 3.105 | 0.061 |

a: Significant models are indicated in Figure 5A,B,E,F

b: Model DF = 1,28 for linear models and 2,27 for quadratic models
