# Supplementary material for: Intraspecific competition counters the effects of elevated and optimal temperatures on phloem-feeding insects in tropical and temperate rice
Source: PLoS One. 2020 Oct 6;15(10):e0240130. doi: 10.1371/journal.pone.0240130 (PMC7538200; doi:10.1371/journal.pone.0240130)
Supplement: S9 Table — (DOCX) [file pone.0240130.s009.docx]

**Table S9. Best fit models to describe the relation between nymph densities and plant weight loss for two rice varieties at constant temperatures of 25°C, 30°C and 35°C**

| Species | Variety | Temperature (°C) | Model^a^ | Constant | B1 | R^2^ | F-value^b^ | P-value |
| --- | --- | --- | --- | --- | --- | --- | --- | --- |
| BPH | IR22 | 25 | Linear | 0.045 | 0.001 | 0.002 | 0.044 | 0.836 |
| BPH | IR22 | 30 | Linear | 0.087 | 0.003 | 0.063 | 1.554 | 0.225 |
| BPH | IR22 | 35 | Linear | 0.088 | -0.003 | 0.324 | 11.043 | 0.003 |
| BPH | T65 | 25 | Linear | 0.080 | 0.001 | 0.010 | 0.228 | 0.637 |
| BPH | T65 | 30 | Linear | 0.126 | -0.002 | 0.010 | 0.242 | 0.627 |
| BPH | T65 | 35 | Quadratic | -0.077 | 0.018 | 0.488 | 10.486 | 0.001 |
| WBPH | IR22 | 25 | Linear | 0.136 | -0.001 | 0.005 | 0.114 | 0.736 |
| WBPH | IR22 | 30 | Linear | 0.120 | 0.002 | 0.044 | 1.051 | 0.316 |
| WBPH | IR22 | 35 | Linear | 0.093 | -0.002 | 0.071 | 1.746 | 0.199 |
| WBPH | T65 | 25 | Linear | 0.096 | 0.003 | 0.047 | 1.130 | 0.299 |
| WBPH | T65 | 30 | Linear | 0.046 | 0.002 | 0.012 | 0.280 | 0.602 |
| WBPH | T65 | 35 | Linear | 0.063 | -0.001 | 0.04 | 0.965 | 0.336 |

a: Significant models are indicated in Figure 5C,D,G,H

b: Model DF = 1,23 for linear models and 2,22 for quadratic models
