# Supplementary material for: Intraspecific competition counters the effects of elevated and optimal temperatures on phloem-feeding insects in tropical and temperate rice
Source: PLoS One. 2020 Oct 6;15(10):e0240130. doi: 10.1371/journal.pone.0240130 (PMC7538200; doi:10.1371/journal.pone.0240130)
Supplement: S10 Table — (DOCX) [file pone.0240130.s010.docx]

**Table S10. Data for plant biomass gain at 25 and 30°C**

| Temperature (oC) | Day | Run | IR22 biomass (g) | T65 biomass (g) | Temperature (oC) | Day | Run | IR22 biomass (g) | T65 biomass (g) |
| --- | --- | --- | --- | --- | --- | --- | --- | --- | --- |
| 25 | 5 | 1 | 0.005 | 0.003 | 30 | 5 | 1 | 0.004 | 0.002 |
| 25 | 5 | 2 | 0.001 | 0.004 | 30 | 5 | 2 | 0.006 | 0.001 |
| 25 | 5 | 3 | 0.004 | 0.004 | 30 | 5 | 3 | 0.002 | 0.002 |
| 25 | 5 | 4 | 0.004 | 0.001 | 30 | 5 | 4 | 0.003 | 0.006 |
| 25 | 7 | 1 | 0.004 | 0.004 | 30 | 7 | 1 | 0.009 | 0.005 |
| 25 | 7 | 2 | 0.003 | 0.007 | 30 | 7 | 2 | 0.007 | 0.008 |
| 25 | 7 | 3 | 0.005 | 0.003 | 30 | 7 | 3 | 0.008 | 0.008 |
| 25 | 7 | 4 | 0.010 | 0.005 | 30 | 7 | 4 | 0.007 | 0.003 |
| 25 | 9 | 1 | 0.007 | 0.014 | 30 | 9 | 1 | 0.007 | 0.004 |
| 25 | 9 | 2 | 0.006 | 0.003 | 30 | 9 | 2 | 0.010 | 0.004 |
| 25 | 9 | 3 | 0.001 | 0.012 | 30 | 9 | 3 | 0.007 | 0.006 |
| 25 | 9 | 4 | 0.008 | 0.006 | 30 | 9 | 4 | 0.007 | 0.005 |
| 25 | 11 | 1 | 0.005 | 0.009 | 30 | 11 | 1 | 0.014 | 0.012 |
| 25 | 11 | 2 | 0.008 | 0.015 | 30 | 11 | 2 | 0.011 | 0.011 |
| 25 | 11 | 3 | 0.005 | 0.010 | 30 | 11 | 3 | 0.011 | 0.006 |
| 25 | 11 | 4 | 0.006 | 0.004 | 30 | 11 | 4 | 0.009 | 0.007 |
| 25 | 13 | 1 | 0.005 | 0.007 | 30 | 13 | 1 | 0.013 | 0.011 |
| 25 | 13 | 2 | 0.005 | 0.011 | 30 | 13 | 2 | 0.004 | 0.010 |
| 25 | 13 | 3 | 0.006 | 0.006 | 30 | 13 | 3 | 0.011 | 0.010 |
| 25 | 13 | 4 | 0.011 | 0.014 | 30 | 13 | 4 | 0.017 | 0.008 |
| 25 | 15 | 1 | 0.006 | 0.002 | 30 | 15 | 1 | 0.014 | 0.016 |
| 25 | 15 | 2 | 0.006 | 0.010 | 30 | 15 | 2 | 0.015 | 0.004 |
| 25 | 15 | 3 | 0.004 | 0.016 | 30 | 15 | 3 | 0.016 | 0.012 |
| 25 | 15 | 4 | 0.007 | 0.006 | 30 | 15 | 4 | 0.017 | 0.011 |
| 25 | 17 | 1 | 0.010 | 0.015 | 30 | 17 | 1 | 0.019 | 0.020 |
| 25 | 17 | 2 | 0.010 | 0.010 | 30 | 17 | 2 | 0.017 | 0.016 |
| 25 | 17 | 3 | 0.014 | 0.022 | 30 | 17 | 3 | 0.026 | 0.014 |
| 25 | 17 | 4 | 0.020 | 0.016 | 30 | 17 | 4 | 0.019 | 0.022 |
| 25 | 18 | 1 | 0.011 | 0.015 | 30 | 18 | 1 | 0.022 | 0.014 |
| 25 | 18 | 2 | 0.008 | 0.010 | 30 | 18 | 2 | 0.017 | 0.017 |
| 25 | 18 | 3 | 0.009 | 0.014 | 30 | 18 | 3 | 0.022 | 0.019 |
| 25 | 18 | 4 | 0.018 | 0.018 | 30 | 18 | 4 | 0.018 | 0.019 |
| 25 | 19 | 1 | 0.016 | 0.019 | 30 | 19 | 1 | 0.018 | 0.018 |
| 25 | 19 | 2 | 0.007 | 0.011 | 30 | 19 | 2 | 0.026 | 0.019 |
| 25 | 19 | 3 | 0.008 | 0.004 | 30 | 19 | 3 | 0.020 | 0.015 |
| 25 | 19 | 4 | 0.006 | 0.022 | 30 | 19 | 4 | 0.020 | 0.018 |
| 25 | 20 | 1 | 0.013 | 0.016 | 30 | 20 | 1 | 0.017 | 0.022 |
| 25 | 20 | 2 | 0.010 | 0.018 | 30 | 20 | 2 | 0.023 | 0.022 |
| 25 | 20 | 3 | 0.022 | 0.021 | 30 | 20 | 3 | 0.021 | 0.027 |
| 25 | 20 | 4 | 0.009 | 0.019 | 30 | 20 | 4 | 0.019 | 0.027 |
| 25 | 21 | 1 | 0.014 | 0.012 | 30 | 21 | 1 | 0.030 | 0.020 |
| 25 | 21 | 2 | 0.018 | 0.011 | 30 | 21 | 2 | 0.029 | 0.019 |
| 25 | 21 | 3 | 0.013 | 0.013 | 30 | 21 | 3 | 0.029 | 0.025 |
| 25 | 21 | 4 | 0.008 | 0.025 | 30 | 21 | 4 | 0.024 | 0.023 |
| 25 | 22 | 1 | 0.032 | 0.023 | 30 | 22 | 1 | 0.027 | 0.025 |
| 25 | 22 | 2 | 0.017 | 0.015 | 30 | 22 | 2 | 0.038 | 0.031 |
| 25 | 22 | 3 | 0.016 | 0.017 | 30 | 22 | 3 | 0.026 | 0.029 |
| 25 | 22 | 4 | 0.018 | 0.031 | 30 | 22 | 4 | 0.035 | 0.037 |
| 25 | 23 | 1 | 0.036 | 0.027 | 30 | 23 | 1 | 0.028 | 0.016 |
| 25 | 23 | 2 | 0.027 | 0.026 | 30 | 23 | 2 | 0.024 | 0.035 |
| 25 | 23 | 3 | 0.013 | 0.027 | 30 | 23 | 3 | 0.027 | 0.022 |
| 25 | 23 | 4 | 0.042 | 0.025 | 30 | 23 | 4 | 0.028 | 0.027 |
| 25 | 24 | 1 | 0.031 | 0.037 | 30 | 24 | 1 | 0.028 | 0.035 |
| 25 | 24 | 2 | 0.018 | 0.032 | 30 | 24 | 2 | 0.032 | 0.037 |
| 25 | 24 | 3 | 0.020 | 0.028 | 30 | 24 | 3 | 0.031 | 0.025 |
| 25 | 24 | 4 | 0.017 | 0.017 | 30 | 24 | 4 | 0.049 | 0.027 |
| 25 | 25 | 1 | 0.039 | 0.081 | 30 | 25 | 1 | 0.028 | 0.043 |
| 25 | 25 | 2 | 0.010 | 0.033 | 30 | 25 | 2 | 0.035 | 0.035 |
| 25 | 25 | 3 | 0.018 | 0.017 | 30 | 25 | 3 | 0.025 | 0.032 |
| 25 | 25 | 4 | 0.033 | 0.027 | 30 | 25 | 4 | 0.036 | 0.036 |
| 25 | 27 | 1 | 0.018 | 0.053 | 30 | 27 | 1 | 0.033 | 0.027 |
| 25 | 27 | 2 | 0.020 | 0.033 | 30 | 27 | 2 | 0.044 | 0.018 |
| 25 | 27 | 3 | 0.034 | 0.009 | 30 | 27 | 3 | 0.045 | 0.023 |
| 25 | 27 | 4 | 0.013 | 0.055 | 30 | 27 | 4 | 0.044 | 0.036 |
| 25 | 29 | 1 | 0.040 | 0.049 | 30 | 29 | 1 | 0.044 | 0.040 |
| 25 | 29 | 2 | 0.037 | 0.050 | 30 | 29 | 2 | 0.056 | 0.037 |
| 25 | 29 | 3 | 0.031 | 0.023 | 30 | 29 | 3 | 0.042 | 0.038 |
| 25 | 29 | 4 | 0.024 | 0.042 | 30 | 29 | 4 | 0.057 | 0.046 |
| 25 | 31 | 1 | 0.050 | 0.050 | 30 | 31 | 1 | 0.048 | 0.055 |
| 25 | 31 | 2 | 0.040 | 0.038 | 30 | 31 | 2 | 0.054 | 0.045 |
| 25 | 31 | 3 | 0.047 | 0.021 | 30 | 31 | 3 | 0.067 | 0.046 |
| 25 | 31 | 4 | 0.043 | 0.050 | 30 | 31 | 4 | 0.048 | 0.035 |
| 25 | 33 | 1 | 0.065 | 0.027 | 30 | 33 | 1 | 0.062 | 0.046 |
| 25 | 33 | 2 | 0.059 | 0.026 | 30 | 33 | 2 | 0.060 | 0.044 |
| 25 | 33 | 3 | 0.027 | 0.026 | 30 | 33 | 3 | 0.052 | 0.043 |
| 25 | 33 | 4 | 0.020 | 0.061 | 30 | 33 | 4 | 0.030 | 0.066 |
| 25 | 35 | 1 | 0.054 | 0.079 | 30 | 35 | 1 | 0.114 | 0.072 |
| 25 | 35 | 2 | 0.056 | 0.038 | 30 | 35 | 2 | 0.085 | 0.087 |
| 25 | 35 | 3 | 0.020 | 0.058 | 30 | 35 | 3 | 0.056 | 0.048 |
| 25 | 35 | 4 | 0.051 | 0.019 | 30 | 35 | 4 | 0.047 | 0.089 |
| 25 | 37 | 1 | 0.054 | 0.040 | 30 | 37 | 1 | 0.075 | 0.049 |
| 25 | 37 | 2 | 0.040 | 0.075 | 30 | 37 | 2 | 0.059 | 0.038 |
| 25 | 37 | 3 | 0.071 | 0.051 | 30 | 37 | 3 | 0.070 | 0.023 |
| 25 | 37 | 4 | 0.055 | 0.081 | 30 | 37 | 4 | 0.045 | 0.071 |
| 25 | 39 | 1 | 0.036 | 0.060 | 30 | 39 | 1 | 0.084 | 0.060 |
| 25 | 39 | 2 | 0.045 | 0.063 | 30 | 39 | 2 | 0.079 | 0.071 |
| 25 | 39 | 3 | 0.048 | 0.052 | 30 | 39 | 3 | 0.074 | 0.063 |
| 25 | 39 | 4 | 0.053 | 0.099 | 30 | 39 | 4 | 0.082 | 0.098 |
