# Supplementary material for: Intraspecific competition counters the effects of elevated and optimal temperatures on phloem-feeding insects in tropical and temperate rice
Source: PLoS One. 2020 Oct 6;15(10):e0240130. doi: 10.1371/journal.pone.0240130 (PMC7538200; doi:10.1371/journal.pone.0240130)
Supplement: S11 Table — (DOCX) [file pone.0240130.s011.docx]

**Table S11. Data from oviposition experiments** (BPH = brown planthopper; WBPH = whitebacked planthopper)

| Temperature (°C) | Planthopper species | Variety | Density (No. per plant) | Run | Total batches | Total eggs | No. of survivors | Planthopper dry weight (mg) | Plant dry weight (g) |
| --- | --- | --- | --- | --- | --- | --- | --- | --- | --- |
| 25 | BPH | IR22 | 1 | 1 | 19.00 | 42.00 | 1.00 | 0.91 | 0.053 |
| 25 | BPH | IR22 | 1 | 2 | 28.00 | 52.00 | 1.00 | 0.67 | 0.038 |
| 25 | BPH | IR22 | 1 | 3 | 28.00 | 112.00 | 1.00 | 1.21 | 0.056 |
| 25 | BPH | IR22 | 1 | 4 | 12.00 | 39.00 | 1.00 | 1.00 | 0.036 |
| 25 | BPH | IR22 | 1 | 5 | 39.00 | 89.00 | 1.00 | 0.96 | 0.051 |
| 25 | BPH | IR22 | 2 | 1 | 46.00 | 125.00 | 2.00 | 1.88 | 0.047 |
| 25 | BPH | IR22 | 2 | 2 | 58.00 | 94.00 | 2.00 | 2.15 | 0.042 |
| 25 | BPH | IR22 | 2 | 3 | 58.00 | 142.00 | 2.00 | 1.34 | 0.049 |
| 25 | BPH | IR22 | 2 | 4 | 31.00 | 98.00 | 2.00 | 2.07 | 0.049 |
| 25 | BPH | IR22 | 2 | 5 | 37.00 | 78.00 | 2.00 | 2.20 | 0.058 |
| 25 | BPH | IR22 | 4 | 1 | 47.00 | 123.00 | 3.00 | 2.56 | 0.049 |
| 25 | BPH | IR22 | 4 | 2 | 152.00 | 229.00 | 4.00 | 3.34 | 0.070 |
| 25 | BPH | IR22 | 4 | 3 | 72.00 | 281.00 | 4.00 | 4.21 | 0.061 |
| 25 | BPH | IR22 | 4 | 4 | 38.00 | 145.00 | 2.00 | 1.56 | 0.056 |
| 25 | BPH | IR22 | 4 | 5 | 84.00 | 173.00 | 3.00 | 2.57 | 0.053 |
| 25 | BPH | IR22 | 6 | 1 | 77.00 | 188.00 | 3.00 | 1.80 | 0.041 |
| 25 | BPH | IR22 | 6 | 2 | 162.00 | 270.00 | 6.00 | 6.32 | 0.047 |
| 25 | BPH | IR22 | 6 | 3 | 102.00 | 317.00 | 5.00 | 4.23 | 0.043 |
| 25 | BPH | IR22 | 6 | 4 | 125.00 | 461.00 | 5.00 | 5.80 | 0.070 |
| 25 | BPH | IR22 | 6 | 5 | 90.00 | 186.00 | 5.00 | 4.54 | 0.047 |
| 25 | BPH | IR22 | 8 | 1 | 66.00 | 254.00 | 5.00 | 3.62 | 0.043 |
| 25 | BPH | IR22 | 8 | 2 | 140.00 | 537.00 | 6.00 | 5.85 | 0.055 |
| 25 | BPH | IR22 | 8 | 3 | 128.00 | 446.00 | 7.00 | 5.92 | 0.041 |
| 25 | BPH | IR22 | 8 | 4 | 129.00 | 435.00 | 7.00 | 6.84 | 0.046 |
| 25 | BPH | IR22 | 8 | 5 | 134.00 | 252.00 | 6.00 | 5.37 | 0.056 |
| 25 | BPH | IR22 | 10 | 1 | 158.00 | 464.00 | 10.00 | 6.87 | 0.041 |
| 25 | BPH | IR22 | 10 | 2 | 214.00 | 650.00 | 8.00 | 8.23 | 0.050 |
| 25 | BPH | IR22 | 10 | 3 | 201.00 | 544.00 | 8.00 | 6.75 | 0.049 |
| 25 | BPH | IR22 | 10 | 4 | 177.00 | 648.00 | 7.00 | 6.53 | 0.065 |
| 25 | BPH | IR22 | 10 | 5 | 150.00 | 242.00 | 9.00 | 7.22 | 0.054 |
| 25 | BPH | IR22 | 12 | 1 | 138.00 | 403.00 | 11.00 | 6.06 | 0.040 |
| 25 | BPH | IR22 | 12 | 2 | 253.00 | 745.00 | 11.00 | 9.26 | 0.047 |
| 25 | BPH | IR22 | 12 | 3 | 215.00 | 519.00 | 10.00 | 8.06 | 0.062 |
| 25 | BPH | IR22 | 12 | 4 | 146.00 | 484.00 | 10.00 | 8.17 | 0.055 |
| 25 | BPH | IR22 | 12 | 5 | 163.00 | 465.00 | 10.00 | 9.63 | 0.055 |
| 25 | BPH | T65 | 1 | 1 | 36.00 | 63.00 | 1.00 | 0.88 | 0.047 |
| 25 | BPH | T65 | 1 | 2 | 32.00 | 66.00 | 1.00 | 1.14 | 0.050 |
| 25 | BPH | T65 | 1 | 3 | 0.00 | 0.00 | 1.00 | 0.45 | 0.088 |
| 25 | BPH | T65 | 1 | 4 | 1.00 | 5.00 | 0.00 | 0.00 | 0.052 |
| 25 | BPH | T65 | 1 | 5 | 30.00 | 53.00 | 0.00 | 0.00 | 0.056 |
| 25 | BPH | T65 | 2 | 1 | 29.00 | 79.00 | 2.00 | 1.70 | 0.048 |
| 25 | BPH | T65 | 2 | 2 | 77.00 | 188.00 | 2.00 | 2.93 | 0.041 |
| 25 | BPH | T65 | 2 | 3 | 44.00 | 137.00 | 2.00 | 2.48 | 0.036 |
| 25 | BPH | T65 | 2 | 4 | 34.00 | 85.00 | 1.00 | 1.72 | 0.059 |
| 25 | BPH | T65 | 2 | 5 | 99.00 | 192.00 | 2.00 | 2.09 | 0.050 |
| 25 | BPH | T65 | 4 | 1 | 52.00 | 104.00 | 4.00 | 3.77 | 0.050 |
| 25 | BPH | T65 | 4 | 2 | 146.00 | 262.00 | 4.00 | 4.89 | 0.050 |
| 25 | BPH | T65 | 4 | 3 | 65.00 | 152.00 | 4.00 | 4.47 | 0.041 |
| 25 | BPH | T65 | 4 | 4 | 74.00 | 322.00 | 3.00 | 4.70 | 0.059 |
| 25 | BPH | T65 | 4 | 5 | 64.00 | 101.00 | 2.00 | 1.63 | 0.057 |
| 25 | BPH | T65 | 6 | 1 | 67.00 | 110.00 | 5.00 | 4.32 | 0.053 |
| 25 | BPH | T65 | 6 | 2 | 157.00 | 412.00 | 6.00 | 5.87 | 0.055 |
| 25 | BPH | T65 | 6 | 3 | 101.00 | 251.00 | 4.00 | 3.71 | 0.051 |
| 25 | BPH | T65 | 6 | 4 | 112.00 | 272.00 | 6.00 | 5.16 | 0.060 |
| 25 | BPH | T65 | 6 | 5 | 65.00 | 118.00 | 3.00 | 2.98 | 0.053 |
| 25 | BPH | T65 | 8 | 1 | 122.00 | 216.00 | 8.00 | 6.40 | 0.064 |
| 25 | BPH | T65 | 8 | 2 | 102.00 | 185.00 | 5.00 | 4.85 | 0.055 |
| 25 | BPH | T65 | 8 | 3 | 162.00 | 382.00 | 7.00 | 5.72 | 0.064 |
| 25 | BPH | T65 | 8 | 4 | 173.00 | 584.00 | 7.00 | 8.52 | 0.064 |
| 25 | BPH | T65 | 8 | 5 | 42.00 | 51.00 | 3.00 | 3.07 | 0.056 |
| 25 | BPH | T65 | 10 | 1 | 121.00 | 271.00 | 6.00 | 3.84 | 0.040 |
| 25 | BPH | T65 | 10 | 2 | 129.00 | 300.00 | 8.00 | 9.14 | 0.036 |
| 25 | BPH | T65 | 10 | 3 | 92.00 | 188.00 | 7.00 | 6.65 | 0.044 |
| 25 | BPH | T65 | 10 | 4 | 95.00 | 356.00 | 6.00 | 6.08 | 0.054 |
| 25 | BPH | T65 | 10 | 5 | 99.00 | 176.00 | 7.00 | 7.23 | 0.060 |
| 25 | BPH | T65 | 12 | 1 | 140.00 | 401.00 | 11.00 | 7.70 | 0.035 |
| 25 | BPH | T65 | 12 | 2 | 229.00 | 1051.00 | 12.00 | 13.11 | 0.039 |
| 25 | BPH | T65 | 12 | 3 | 184.00 | 453.00 | 11.00 | 7.34 | 0.044 |
| 25 | BPH | T65 | 12 | 4 | 172.00 | 364.00 | 8.00 | 8.71 | 0.049 |
| 25 | BPH | T65 | 12 | 5 | 148.00 | 218.00 | 6.00 | 5.35 | 0.031 |
| 30 | BPH | IR22 | 1 | 1 | 39.00 | 61.00 | 1.00 | 0.67 | 0.034 |
| 30 | BPH | IR22 | 1 | 2 | 24.00 | 37.00 | 1.00 | 0.78 | 0.053 |
| 30 | BPH | IR22 | 1 | 3 | 33.00 | 125.00 | 1.00 | 1.47 | 0.033 |
| 30 | BPH | IR22 | 1 | 4 | 22.00 | 47.00 | 1.00 | 0.80 | 0.038 |
| 30 | BPH | IR22 | 1 | 5 | 7.00 | 14.00 | 0.00 | 0.00 | 0.033 |
| 30 | BPH | IR22 | 2 | 1 | 47.00 | 87.00 | 2.00 | 1.32 | 0.042 |
| 30 | BPH | IR22 | 2 | 2 | 86.00 | 203.00 | 2.00 | 2.47 | 0.041 |
| 30 | BPH | IR22 | 2 | 3 | 95.00 | 299.00 | 2.00 | 2.59 | 0.050 |
| 30 | BPH | IR22 | 2 | 4 | 26.00 | 91.00 | 0.00 | 0.00 | 0.037 |
| 30 | BPH | IR22 | 2 | 5 | 79.00 | 141.00 | 2.00 | 1.82 | 0.052 |
| 30 | BPH | IR22 | 4 | 1 | 85.00 | 202.00 | 3.00 | 2.63 | 0.039 |
| 30 | BPH | IR22 | 4 | 2 | 132.00 | 431.00 | 4.00 | 3.82 | 0.048 |
| 30 | BPH | IR22 | 4 | 3 | 77.00 | 192.00 | 4.00 | 3.00 | 0.054 |
| 30 | BPH | IR22 | 4 | 4 | 66.00 | 240.00 | 3.00 | 3.16 | 0.033 |
| 30 | BPH | IR22 | 4 | 5 | 129.00 | 276.00 | 3.00 | 2.47 | 0.044 |
| 30 | BPH | IR22 | 6 | 1 | 66.00 | 140.00 | 4.00 | 2.64 | 0.033 |
| 30 | BPH | IR22 | 6 | 2 | 156.00 | 401.00 | 6.00 | 5.51 | 0.054 |
| 30 | BPH | IR22 | 6 | 3 | 154.00 | 334.00 | 6.00 | 4.45 | 0.055 |
| 30 | BPH | IR22 | 6 | 4 | 107.00 | 330.00 | 3.00 | 4.41 | 0.057 |
| 30 | BPH | IR22 | 6 | 5 | 110.00 | 224.00 | 5.00 | 2.96 | 0.054 |
| 30 | BPH | IR22 | 8 | 1 | 71.00 | 159.00 | 5.00 | 3.20 | 0.044 |
| 30 | BPH | IR22 | 8 | 2 | 161.00 | 449.00 | 7.00 | 5.88 | 0.064 |
| 30 | BPH | IR22 | 8 | 3 | 165.00 | 467.00 | 7.00 | 5.46 | 0.046 |
| 30 | BPH | IR22 | 8 | 4 | 140.00 | 464.00 | 2.00 | 2.88 | 0.042 |
| 30 | BPH | IR22 | 8 | 5 | 99.00 | 222.00 | 2.00 | 1.52 | 0.053 |
| 30 | BPH | IR22 | 10 | 1 | 100.00 | 260.00 | 6.00 | 3.66 | 0.039 |
| 30 | BPH | IR22 | 10 | 2 | 248.00 | 897.00 | 9.00 | 8.76 | 0.040 |
| 30 | BPH | IR22 | 10 | 3 | 235.00 | 589.00 | 8.00 | 5.48 | 0.047 |
| 30 | BPH | IR22 | 10 | 4 | 224.00 | 797.00 | 8.00 | 7.27 | 0.047 |
| 30 | BPH | IR22 | 10 | 5 | 147.00 | 307.00 | 4.00 | 3.06 | 0.059 |
| 30 | BPH | IR22 | 12 | 1 | 92.00 | 254.00 | 5.00 | 3.41 | 0.035 |
| 30 | BPH | IR22 | 12 | 2 | 243.00 | 754.00 | 12.00 | 8.64 | 0.038 |
| 30 | BPH | IR22 | 12 | 3 | 279.00 | 945.00 | 12.00 | 8.15 | 0.038 |
| 30 | BPH | IR22 | 12 | 4 | 212.00 | 722.00 | 5.00 | 5.74 | 0.044 |
| 30 | BPH | IR22 | 12 | 5 | 143.00 | 245.00 | 1.00 | 0.70 | 0.060 |
| 30 | BPH | T65 | 1 | 1 | 30.00 | 68.00 | 1.00 | 1.12 | 0.029 |
| 30 | BPH | T65 | 1 | 2 | 43.00 | 125.00 | 1.00 | 1.84 | 0.045 |
| 30 | BPH | T65 | 1 | 3 | 30.00 | 82.00 | 1.00 | 0.91 | 0.042 |
| 30 | BPH | T65 | 1 | 4 | 42.00 | 113.00 | 1.00 | 1.32 | 0.040 |
| 30 | BPH | T65 | 1 | 5 | 48.00 | 92.00 | 1.00 | 1.11 | 0.061 |
| 30 | BPH | T65 | 2 | 1 | 46.00 | 82.00 | 1.00 | 1.03 | 0.038 |
| 30 | BPH | T65 | 2 | 2 | 103.00 | 193.00 | 2.00 | 2.19 | 0.045 |
| 30 | BPH | T65 | 2 | 3 | 61.00 | 88.00 | 0.00 | 0.00 | 0.044 |
| 30 | BPH | T65 | 2 | 4 | 64.00 | 128.00 | 0.00 | 0.00 | 0.045 |
| 30 | BPH | T65 | 2 | 5 | 76.00 | 172.00 | 2.00 | 1.61 | 0.059 |
| 30 | BPH | T65 | 4 | 1 | 88.00 | 153.00 | 4.00 | 3.27 | 0.051 |
| 30 | BPH | T65 | 4 | 2 | 131.00 | 281.00 | 3.00 | 2.99 | 0.064 |
| 30 | BPH | T65 | 4 | 3 | 107.00 | 187.00 | 3.00 | 3.43 | 0.056 |
| 30 | BPH | T65 | 4 | 4 | 12.00 | 32.00 | 0.00 | 0.00 | 0.054 |
| 30 | BPH | T65 | 4 | 5 | 92.00 | 118.00 | 1.00 | 0.97 | 0.056 |
| 30 | BPH | T65 | 6 | 1 | 88.00 | 211.00 | 5.00 | 3.98 | 0.044 |
| 30 | BPH | T65 | 6 | 2 | 159.00 | 319.00 | 5.00 | 4.78 | 0.056 |
| 30 | BPH | T65 | 6 | 3 | 220.00 | 459.00 | 6.00 | 4.21 | 0.042 |
| 30 | BPH | T65 | 6 | 4 | 94.00 | 255.00 | 2.00 | 2.20 | 0.040 |
| 30 | BPH | T65 | 6 | 5 | 132.00 | 206.00 | 3.00 | 2.23 | 0.034 |
| 30 | BPH | T65 | 8 | 1 | 61.00 | 118.00 | 6.00 | 3.76 | 0.050 |
| 30 | BPH | T65 | 8 | 2 | 166.00 | 331.00 | 6.00 | 5.53 | 0.041 |
| 30 | BPH | T65 | 8 | 3 | 166.00 | 385.00 | 6.00 | 3.59 | 0.055 |
| 30 | BPH | T65 | 8 | 4 | 162.00 | 502.00 | 6.00 | 8.34 | 0.051 |
| 30 | BPH | T65 | 8 | 5 | 146.00 | 251.00 | 4.00 | 3.56 | 0.049 |
| 30 | BPH | T65 | 10 | 1 | 101.00 | 189.00 | 7.00 | 4.39 | 0.042 |
| 30 | BPH | T65 | 10 | 2 | 222.00 | 369.00 | 9.00 | 10.15 | 0.051 |
| 30 | BPH | T65 | 10 | 3 | 214.00 | 407.00 | 8.00 | 5.34 | 0.048 |
| 30 | BPH | T65 | 10 | 4 | 174.00 | 540.00 | 4.00 | 5.39 | 0.041 |
| 30 | BPH | T65 | 10 | 5 | 173.00 | 293.00 | 7.00 | 5.97 | 0.035 |
| 30 | BPH | T65 | 12 | 1 | 120.00 | 261.00 | 10.00 | 6.03 | 0.049 |
| 30 | BPH | T65 | 12 | 2 | 225.00 | 494.00 | 11.00 | 10.86 | 0.039 |
| 30 | BPH | T65 | 12 | 3 | 70.00 | 128.00 | 9.00 | 7.62 | 0.074 |
| 30 | BPH | T65 | 12 | 4 | 350.00 | 375.00 | 7.00 | 7.54 | 0.050 |
| 30 | BPH | T65 | 12 | 5 | 108.00 | 184.00 | 3.00 | 2.87 | 0.056 |
| 35 | BPH | IR22 | 1 | 1 | 1.00 | 57.00 | 1.00 |  | 0.055 |
| 35 | BPH | IR22 | 1 | 2 | 2.00 | 52.00 | 0.00 |  | 0.053 |
| 35 | BPH | IR22 | 1 | 3 | 3.00 | 59.00 | 1.00 |  | 0.065 |
| 35 | BPH | IR22 | 1 | 4 | 4.00 | 56.00 | 0.00 |  | 0.056 |
| 35 | BPH | IR22 | 1 | 5 | 5.00 | 52.50 | 0.50 |  | 0.039 |
| 35 | BPH | IR22 | 2 | 1 | 1.00 | 82.00 | 1.00 |  | 0.061 |
| 35 | BPH | IR22 | 2 | 2 | 2.00 | 108.00 | 1.00 |  | 0.056 |
| 35 | BPH | IR22 | 2 | 3 | 3.00 | 90.00 | 1.00 |  | 0.056 |
| 35 | BPH | IR22 | 2 | 4 | 4.00 | 114.00 | 1.00 |  | 0.043 |
| 35 | BPH | IR22 | 2 | 5 | 5.00 | 95.00 | 0.50 |  | 0.044 |
| 35 | BPH | IR22 | 4 | 1 | 1.00 | 213.00 | 3.00 |  | 0.047 |
| 35 | BPH | IR22 | 4 | 2 | 2.00 | 291.00 | 1.00 |  | 0.055 |
| 35 | BPH | IR22 | 4 | 3 | 3.00 | 123.00 | 1.00 |  | 0.059 |
| 35 | BPH | IR22 | 4 | 4 | 4.00 | 118.00 | 2.00 |  | 0.057 |
| 35 | BPH | IR22 | 4 | 5 | 5.00 | 233.50 | 3.00 |  | 0.060 |
| 35 | BPH | IR22 | 6 | 1 | 1.00 | 357.00 | 5.00 |  | 0.072 |
| 35 | BPH | IR22 | 6 | 2 | 2.00 | 366.00 | 3.00 |  | 0.043 |
| 35 | BPH | IR22 | 6 | 3 | 3.00 | 271.00 | 2.00 |  | 0.076 |
| 35 | BPH | IR22 | 6 | 4 | 4.00 | 241.00 | 1.00 |  | 0.030 |
| 35 | BPH | IR22 | 6 | 5 | 5.00 | 292.50 | 3.50 |  | 0.063 |
| 35 | BPH | IR22 | 8 | 1 | 1.00 | 473.00 | 8.00 |  | 0.058 |
| 35 | BPH | IR22 | 8 | 2 | 2.00 | 442.00 | 4.00 |  | 0.064 |
| 35 | BPH | IR22 | 8 | 3 | 3.00 | 346.00 | 7.00 |  | 0.062 |
| 35 | BPH | IR22 | 8 | 4 | 4.00 | 507.00 | 8.00 |  | 0.058 |
| 35 | BPH | IR22 | 8 | 5 | 5.00 | 359.50 | 5.50 |  | 0.059 |
| 35 | BPH | IR22 | 10 | 1 | 1.00 | 515.00 | 8.00 |  | 0.055 |
| 35 | BPH | IR22 | 10 | 2 | 2.00 | 515.00 | 8.00 |  | 0.055 |
| 35 | BPH | IR22 | 10 | 3 | 3.00 | 363.00 | 5.00 |  | 0.078 |
| 35 | BPH | IR22 | 10 | 4 | 4.00 | 458.00 | 7.00 |  | 0.060 |
| 35 | BPH | IR22 | 10 | 5 | 5.00 | 390.00 | 5.50 |  | 0.074 |
| 35 | BPH | IR22 | 12 | 1 | 1.00 | 509.00 | 10.00 |  | 0.049 |
| 35 | BPH | IR22 | 12 | 2 | 2.00 | 555.00 | 7.00 |  | 0.037 |
| 35 | BPH | IR22 | 12 | 3 | 3.00 | 471.00 | 9.00 |  | 0.049 |
| 35 | BPH | IR22 | 12 | 4 | 4.00 | 568.00 | 8.00 |  | 0.046 |
| 35 | BPH | IR22 | 12 | 5 | 5.00 | 534.50 | 8.50 |  | 0.058 |
| 35 | BPH | T65 | 1 | 1 | 1.00 | 70.00 | 1.00 |  | 0.044 |
| 35 | BPH | T65 | 1 | 2 | 2.00 | 62.00 | 1.00 |  | 0.042 |
| 35 | BPH | T65 | 1 | 3 | 3.00 | 45.00 | 0.00 |  | 0.051 |
| 35 | BPH | T65 | 1 | 4 | 4.00 | 61.00 | 1.00 |  | 0.047 |
| 35 | BPH | T65 | 1 | 5 | 5.00 | 38.00 | 1.00 |  | 0.040 |
| 35 | BPH | T65 | 2 | 1 | 1.00 | 141.00 | 2.00 |  | 0.047 |
| 35 | BPH | T65 | 2 | 2 | 2.00 | 132.00 | 1.00 |  | 0.057 |
| 35 | BPH | T65 | 2 | 3 | 3.00 | 101.00 | 2.00 |  | 0.043 |
| 35 | BPH | T65 | 2 | 4 | 4.00 | 52.00 | 1.00 |  | 0.041 |
| 35 | BPH | T65 | 2 | 5 | 5.00 | 150.50 | 2.00 |  | 0.052 |
| 35 | BPH | T65 | 4 | 1 | 1.00 | 159.00 | 1.00 |  | 0.042 |
| 35 | BPH | T65 | 4 | 2 | 2.00 | 59.00 | 1.00 |  | 0.048 |
| 35 | BPH | T65 | 4 | 3 | 3.00 | 175.00 | 1.00 |  | 0.048 |
| 35 | BPH | T65 | 4 | 4 | 4.00 | 178.00 | 3.00 |  | 0.034 |
| 35 | BPH | T65 | 4 | 5 | 5.00 | 186.50 | 2.50 |  | 0.039 |
| 35 | BPH | T65 | 6 | 1 | 1.00 | 355.00 | 4.00 |  | 0.040 |
| 35 | BPH | T65 | 6 | 2 | 2.00 | 213.00 | 3.00 |  | 0.047 |
| 35 | BPH | T65 | 6 | 3 | 3.00 | 388.00 | 5.00 |  | 0.048 |
| 35 | BPH | T65 | 6 | 4 | 4.00 | 296.00 | 5.00 |  | 0.052 |
| 35 | BPH | T65 | 6 | 5 | 5.00 | 273.50 | 3.50 |  | 0.034 |
| 35 | BPH | T65 | 8 | 1 | 1.00 | 277.00 | 4.00 |  | 0.060 |
| 35 | BPH | T65 | 8 | 2 | 2.00 | 339.00 | 5.00 |  | 0.050 |
| 35 | BPH | T65 | 8 | 3 | 3.00 | 216.00 | 2.00 |  | 0.054 |
| 35 | BPH | T65 | 8 | 4 | 4.00 | 449.00 | 7.00 |  | 0.057 |
| 35 | BPH | T65 | 8 | 5 | 5.00 | 370.00 | 4.00 |  | 0.067 |
| 35 | BPH | T65 | 10 | 1 | 1.00 | 385.00 | 6.00 |  | 0.054 |
| 35 | BPH | T65 | 10 | 2 | 2.00 | 392.00 | 7.00 |  | 0.062 |
| 35 | BPH | T65 | 10 | 3 | 3.00 | 322.00 | 5.00 |  | 0.064 |
| 35 | BPH | T65 | 10 | 4 | 4.00 | 367.00 | 6.00 |  | 0.070 |
| 35 | BPH | T65 | 10 | 5 | 5.00 | 352.00 | 6.50 |  | 0.044 |
| 35 | BPH | T65 | 12 | 1 | 1.00 | 334.00 | 7.00 |  | 0.050 |
| 35 | BPH | T65 | 12 | 2 | 2.00 | 442.00 | 8.00 |  | 0.063 |
| 35 | BPH | T65 | 12 | 3 | 3.00 | 421.00 | 4.00 |  | 0.062 |
| 35 | BPH | T65 | 12 | 4 | 4.00 | 431.00 | 8.00 |  | 0.060 |
| 35 | BPH | T65 | 12 | 5 | 5.00 | 338.00 | 7.00 |  | 0.060 |
| 25 | WBPH | IR22 | 1 | 1 | 0.00 | 0.00 | 1.00 | 0.00 | 0.063 |
| 25 | WBPH | IR22 | 1 | 2 | 6.00 | 34.00 | 1.00 | 0.44 | 0.050 |
| 25 | WBPH | IR22 | 1 | 3 | 6.00 | 58.00 | 0.00 | 0.00 | 0.070 |
| 25 | WBPH | IR22 | 1 | 4 | 1.00 | 0.00 | 0.00 | 0.00 | 0.042 |
| 25 | WBPH | IR22 | 1 | 5 | 1.00 | 0.00 | 0.00 | 0.00 | 0.060 |
| 25 | WBPH | IR22 | 2 | 1 | 5.00 | 29.00 | 1.00 | 0.73 | 0.055 |
| 25 | WBPH | IR22 | 2 | 2 | 7.00 | 43.00 | 2.00 | 1.18 | 0.048 |
| 25 | WBPH | IR22 | 2 | 3 | 14.00 | 64.00 | 0.00 | 0.00 | 0.063 |
| 25 | WBPH | IR22 | 2 | 4 | 17.00 | 104.00 | 1.00 | 0.69 | 0.046 |
| 25 | WBPH | IR22 | 2 | 5 | 9.00 | 36.00 | 2.00 | 0.92 | 0.061 |
| 25 | WBPH | IR22 | 4 | 1 | 33.00 | 190.00 | 3.00 | 1.74 | 0.016 |
| 25 | WBPH | IR22 | 4 | 2 | 12.00 | 54.00 | 2.00 | 1.25 | 0.011 |
| 25 | WBPH | IR22 | 4 | 3 | 13.00 | 75.00 | 3.00 | 2.08 | 0.022 |
| 25 | WBPH | IR22 | 4 | 4 | 13.00 | 120.00 | 1.00 | 0.68 | 0.015 |
| 25 | WBPH | IR22 | 4 | 5 | 1.00 | 3.00 | 0.00 | 0.00 | 0.012 |
| 25 | WBPH | IR22 | 6 | 1 | 27.00 | 152.00 | 4.00 | 2.05 | 0.013 |
| 25 | WBPH | IR22 | 6 | 2 | 37.00 | 143.00 | 6.00 | 3.93 | 0.016 |
| 25 | WBPH | IR22 | 6 | 3 | 28.00 | 159.00 | 5.00 | 2.65 | 0.016 |
| 25 | WBPH | IR22 | 6 | 4 | 24.00 | 154.00 | 2.00 | 1.44 | 0.020 |
| 25 | WBPH | IR22 | 6 | 5 | 7.00 | 26.00 | 0.00 | 0.00 | 0.019 |
| 25 | WBPH | IR22 | 8 | 1 | 30.00 | 175.00 | 2.00 | 0.85 | 0.036 |
| 25 | WBPH | IR22 | 8 | 2 | 45.00 | 231.00 | 6.00 | 3.16 | 0.019 |
| 25 | WBPH | IR22 | 8 | 3 | 47.00 | 168.00 | 5.00 | 2.15 | 0.013 |
| 25 | WBPH | IR22 | 8 | 4 | 32.00 | 238.00 | 3.00 | 2.02 | 0.022 |
| 25 | WBPH | IR22 | 8 | 5 | 16.00 | 84.00 | 1.00 | 0.49 | 0.018 |
| 25 | WBPH | IR22 | 10 | 1 | 41.00 | 183.00 | 7.00 | 3.78 | 0.018 |
| 25 | WBPH | IR22 | 10 | 2 | 74.00 | 411.00 | 10.00 | 5.10 | 0.023 |
| 25 | WBPH | IR22 | 10 | 3 | 48.00 | 229.00 | 4.00 | 2.17 | 0.022 |
| 25 | WBPH | IR22 | 10 | 4 | 14.00 | 113.00 | 1.00 | 0.83 | 0.031 |
| 25 | WBPH | IR22 | 10 | 5 | 22.00 | 118.00 | 1.00 | 0.49 | 0.013 |
| 25 | WBPH | IR22 | 12 | 1 | 54.00 | 301.00 | 5.00 | 2.84 | 0.020 |
| 25 | WBPH | IR22 | 12 | 2 | 104.00 | 521.00 | 10.00 | 5.51 | 0.021 |
| 25 | WBPH | IR22 | 12 | 3 | 53.00 | 241.00 | 6.00 | 2.51 | 0.019 |
| 25 | WBPH | IR22 | 12 | 4 | 21.00 | 162.00 | 0.00 | 0.00 | 0.024 |
| 25 | WBPH | IR22 | 12 | 5 | 10.00 | 53.00 | 2.00 | 1.13 | 0.024 |
| 25 | WBPH | T65 | 1 | 1 | 8.00 | 39.00 | 0.00 | 0.00 | 0.024 |
| 25 | WBPH | T65 | 1 | 2 | 18.00 | 83.00 | 1.00 | 1.01 | 0.024 |
| 25 | WBPH | T65 | 1 | 3 | 16.00 | 81.00 | 1.00 | 0.63 | 0.025 |
| 25 | WBPH | T65 | 1 | 4 | 2.00 | 6.00 | 0.00 | 0.00 | 0.025 |
| 25 | WBPH | T65 | 1 | 5 | 10.00 | 34.00 | 0.00 | 0.00 | 0.025 |
| 25 | WBPH | T65 | 2 | 1 | 5.00 | 18.00 | 1.00 | 0.39 | 0.021 |
| 25 | WBPH | T65 | 2 | 2 | 21.00 | 143.00 | 2.00 | 1.07 | 0.016 |
| 25 | WBPH | T65 | 2 | 3 | 16.00 | 67.00 | 0.00 | 0.00 | 0.017 |
| 25 | WBPH | T65 | 2 | 4 | 18.00 | 104.00 | 1.00 | 0.89 | 0.023 |
| 25 | WBPH | T65 | 2 | 5 | 8.00 | 21.00 | 0.00 | 0.00 | 0.021 |
| 25 | WBPH | T65 | 4 | 1 | 18.00 | 75.00 | 3.00 | 1.95 | 0.019 |
| 25 | WBPH | T65 | 4 | 2 | 33.00 | 158.00 | 4.00 | 3.67 | 0.019 |
| 25 | WBPH | T65 | 4 | 3 | 21.00 | 111.00 | 1.00 | 0.66 | 0.019 |
| 25 | WBPH | T65 | 4 | 4 | 20.00 | 113.00 | 0.00 | 0.00 | 0.018 |
| 25 | WBPH | T65 | 4 | 5 | 7.00 | 35.00 | 0.00 | 0.00 | 0.014 |
| 25 | WBPH | T65 | 6 | 1 | 26.00 | 88.00 | 3.00 | 1.99 | 0.019 |
| 25 | WBPH | T65 | 6 | 2 | 40.00 | 226.00 | 5.00 | 3.07 | 0.012 |
| 25 | WBPH | T65 | 6 | 3 | 28.00 | 106.00 | 5.00 | 2.72 | 0.021 |
| 25 | WBPH | T65 | 6 | 4 | 50.00 | 247.00 | 6.00 | 4.85 | 0.021 |
| 25 | WBPH | T65 | 6 | 5 | 51.00 | 124.00 | 2.00 | 1.29 | 0.020 |
| 25 | WBPH | T65 | 8 | 1 | 52.00 | 188.00 | 3.00 | 1.80 | 0.017 |
| 25 | WBPH | T65 | 8 | 2 | 64.00 | 330.00 | 5.00 | 3.43 | 0.023 |
| 25 | WBPH | T65 | 8 | 3 | 70.00 | 229.00 | 4.00 | 1.99 | 0.025 |
| 25 | WBPH | T65 | 8 | 4 | 34.00 | 128.00 | 4.00 | 3.66 | 0.024 |
| 25 | WBPH | T65 | 8 | 5 | 19.00 | 102.00 | 0.00 | 0.00 | 0.020 |
| 25 | WBPH | T65 | 10 | 1 | 54.00 | 223.00 | 6.00 | 3.61 | 0.022 |
| 25 | WBPH | T65 | 10 | 2 | 70.00 | 307.00 | 9.00 | 5.67 | 0.022 |
| 25 | WBPH | T65 | 10 | 3 | 66.00 | 227.00 | 6.00 | 3.46 | 0.023 |
| 25 | WBPH | T65 | 10 | 4 | 41.00 | 204.00 | 5.00 | 4.15 | 0.025 |
| 25 | WBPH | T65 | 10 | 5 | 8.00 | 52.00 | 1.00 | 0.50 | 0.020 |
| 25 | WBPH | T65 | 12 | 1 | 56.00 | 214.00 | 7.00 | 4.75 | 0.024 |
| 25 | WBPH | T65 | 12 | 2 | 80.00 | 298.00 | 9.00 | 6.89 | 0.025 |
| 25 | WBPH | T65 | 12 | 3 | 49.00 | 197.00 | 5.00 | 2.72 | 0.024 |
| 25 | WBPH | T65 | 12 | 4 | 44.00 | 208.00 | 6.00 | 4.41 | 0.025 |
| 25 | WBPH | T65 | 12 | 5 | 8.00 | 39.00 | 0.00 | 0.00 | 0.025 |
| 30 | WBPH | IR22 | 1 | 1 | 5.00 | 17.00 | 0.00 | 0.00 | 0.015 |
| 30 | WBPH | IR22 | 1 | 2 | 0.00 | 0.00 | 1.00 | 0.70 | 0.015 |
| 30 | WBPH | IR22 | 1 | 3 | 6.00 | 31.00 | 1.00 | 0.57 | 0.013 |
| 30 | WBPH | IR22 | 1 | 4 | 18.00 | 100.00 | 1.00 | 0.59 | 0.011 |
| 30 | WBPH | IR22 | 1 | 5 | 2.00 | 8.00 | 0.00 | 0.00 | 0.020 |
| 30 | WBPH | IR22 | 2 | 1 | 3.00 | 6.00 | 0.00 | 0.00 | 0.015 |
| 30 | WBPH | IR22 | 2 | 2 | 16.00 | 60.00 | 2.00 | 1.18 | 0.020 |
| 30 | WBPH | IR22 | 2 | 3 | 4.00 | 8.00 | 0.00 | 0.00 | 0.010 |
| 30 | WBPH | IR22 | 2 | 4 | 1.00 | 0.00 | 0.00 | 0.00 | 0.012 |
| 30 | WBPH | IR22 | 2 | 5 | 1.00 | 0.00 | 0.00 | 0.00 | 0.017 |
| 30 | WBPH | IR22 | 4 | 1 | 8.00 | 27.00 | 0.00 | 0.00 | 0.014 |
| 30 | WBPH | IR22 | 4 | 2 | 14.00 | 39.00 | 0.00 | 0.00 | 0.019 |
| 30 | WBPH | IR22 | 4 | 3 | 26.00 | 106.00 | 2.00 | 0.86 | 0.012 |
| 30 | WBPH | IR22 | 4 | 4 | 24.00 | 135.00 | 4.00 | 2.53 | 0.017 |
| 30 | WBPH | IR22 | 4 | 5 | 26.00 | 84.00 | 0.00 | 0.00 | 0.010 |
| 30 | WBPH | IR22 | 6 | 1 | 25.00 | 86.00 | 0.00 | 0.00 | 0.024 |
| 30 | WBPH | IR22 | 6 | 2 | 17.00 | 51.00 | 2.00 | 1.17 | 0.015 |
| 30 | WBPH | IR22 | 6 | 3 | 26.00 | 92.00 | 3.00 | 1.28 | 0.020 |
| 30 | WBPH | IR22 | 6 | 4 | 41.00 | 149.00 | 3.00 | 2.03 | 0.011 |
| 30 | WBPH | IR22 | 6 | 5 | 12.00 | 41.00 | 0.00 | 0.00 | 0.018 |
| 30 | WBPH | IR22 | 8 | 1 | 18.00 | 87.00 | 0.00 | 0.00 | 0.012 |
| 30 | WBPH | IR22 | 8 | 2 | 62.00 | 343.00 | 6.00 | 3.01 | 0.022 |
| 30 | WBPH | IR22 | 8 | 3 | 28.00 | 96.00 | 1.00 | 0.42 | 0.012 |
| 30 | WBPH | IR22 | 8 | 4 | 34.00 | 148.00 | 2.00 | 1.79 | 0.020 |
| 30 | WBPH | IR22 | 8 | 5 | 16.00 | 42.00 | 0.00 | 0.00 | 0.025 |
| 30 | WBPH | IR22 | 10 | 1 | 22.00 | 119.00 | 0.00 | 0.00 | 0.019 |
| 30 | WBPH | IR22 | 10 | 2 | 43.00 | 223.00 | 6.00 | 3.36 | 0.019 |
| 30 | WBPH | IR22 | 10 | 3 | 34.00 | 104.00 | 0.00 | 0.00 | 0.018 |
| 30 | WBPH | IR22 | 10 | 4 | 42.00 | 187.00 | 6.00 | 3.95 | 0.021 |
| 30 | WBPH | IR22 | 10 | 5 | 16.00 | 55.00 | 0.00 | 0.00 | 0.025 |
| 30 | WBPH | IR22 | 12 | 1 | 43.00 | 200.00 | 0.00 | 0.00 | 0.020 |
| 30 | WBPH | IR22 | 12 | 2 | 80.00 | 325.00 | 5.00 | 2.78 | 0.014 |
| 30 | WBPH | IR22 | 12 | 3 | 87.00 | 287.00 | 1.00 | 1.19 | 0.016 |
| 30 | WBPH | IR22 | 12 | 4 | 28.00 | 141.00 | 2.00 | 1.37 | 0.018 |
| 30 | WBPH | IR22 | 12 | 5 | 19.00 | 73.00 | 0.00 | 0.00 | 0.021 |
| 30 | WBPH | T65 | 1 | 1 | 16.00 | 72.00 | 1.00 | 0.74 | 0.021 |
| 30 | WBPH | T65 | 1 | 2 | 27.00 | 75.00 | 1.00 | 1.09 | 0.026 |
| 30 | WBPH | T65 | 1 | 3 | 0.00 | 0.00 | 0.00 | 0.00 | 0.016 |
| 30 | WBPH | T65 | 1 | 4 | 21.00 | 80.00 | 1.00 | 0.95 | 0.019 |
| 30 | WBPH | T65 | 1 | 5 | 2.00 | 5.00 | 0.00 | 0.00 | 0.018 |
| 30 | WBPH | T65 | 2 | 1 | 6.00 | 22.00 | 0.00 | 0.00 | 0.027 |
| 30 | WBPH | T65 | 2 | 2 | 24.00 | 86.00 | 2.00 | 1.67 | 0.016 |
| 30 | WBPH | T65 | 2 | 3 | 19.00 | 64.00 | 1.00 | 0.29 | 0.012 |
| 30 | WBPH | T65 | 2 | 4 | 39.00 | 177.00 | 2.00 | 1.87 | 0.028 |
| 30 | WBPH | T65 | 2 | 5 | 6.00 | 13.00 | 0.00 | 0.00 | 0.023 |
| 30 | WBPH | T65 | 4 | 1 | 22.00 | 82.00 | 0.00 | 0.00 | 0.025 |
| 30 | WBPH | T65 | 4 | 2 | 58.00 | 207.00 | 3.00 | 2.44 | 0.021 |
| 30 | WBPH | T65 | 4 | 3 | 11.00 | 36.00 | 1.00 | 0.57 | 0.024 |
| 30 | WBPH | T65 | 4 | 4 | 9.00 | 40.00 | 1.00 | 1.14 | 0.015 |
| 30 | WBPH | T65 | 4 | 5 | 13.00 | 49.00 | 1.00 | 0.42 | 0.022 |
| 30 | WBPH | T65 | 6 | 1 | 40.00 | 117.00 | 2.00 | 1.39 | 0.053 |
| 30 | WBPH | T65 | 6 | 2 | 59.00 | 197.00 | 4.00 | 2.96 | 0.038 |
| 30 | WBPH | T65 | 6 | 3 | 48.00 | 153.00 | 3.00 | 1.54 | 0.056 |
| 30 | WBPH | T65 | 6 | 4 | 77.00 | 375.00 | 5.00 | 3.64 | 0.036 |
| 30 | WBPH | T65 | 6 | 5 | 10.00 | 43.00 | 1.00 | 0.60 | 0.051 |
| 30 | WBPH | T65 | 8 | 1 | 42.00 | 152.00 | 4.00 | 2.46 | 0.047 |
| 30 | WBPH | T65 | 8 | 2 | 52.00 | 229.00 | 6.00 | 4.31 | 0.042 |
| 30 | WBPH | T65 | 8 | 3 | 17.00 | 39.00 | 1.00 | 0.60 | 0.049 |
| 30 | WBPH | T65 | 8 | 4 | 87.00 | 381.00 | 3.00 | 3.32 | 0.049 |
| 30 | WBPH | T65 | 8 | 5 | 13.00 | 45.00 | 2.00 | 0.92 | 0.058 |
| 30 | WBPH | T65 | 10 | 1 | 30.00 | 101.00 | 4.00 | 2.63 | 0.049 |
| 30 | WBPH | T65 | 10 | 2 | 23.00 | 69.00 | 9.00 | 6.73 | 0.070 |
| 30 | WBPH | T65 | 10 | 3 | 37.00 | 110.00 | 2.00 | 1.23 | 0.061 |
| 30 | WBPH | T65 | 10 | 4 | 41.00 | 214.00 | 4.00 | 3.38 | 0.056 |
| 30 | WBPH | T65 | 10 | 5 | 11.00 | 23.00 | 0.00 | 0.00 | 0.053 |
| 30 | WBPH | T65 | 12 | 1 | 32.00 | 88.00 | 4.00 | 2.29 | 0.041 |
| 30 | WBPH | T65 | 12 | 2 | 72.00 | 284.00 | 5.00 | 3.38 | 0.047 |
| 30 | WBPH | T65 | 12 | 3 | 47.00 | 144.00 | 3.00 | 1.44 | 0.043 |
| 30 | WBPH | T65 | 12 | 4 | 80.00 | 289.00 | 9.00 | 6.67 | 0.070 |
| 30 | WBPH | T65 | 12 | 5 | 19.00 | 86.00 | 0.00 | 0.00 | 0.047 |
| 35 | WBPH | IR22 | 1 | 1 | 1.00 | 79.00 | 1.00 |  | 0.043 |
| 35 | WBPH | IR22 | 1 | 2 | 2.00 | 72.00 | 1.00 |  | 0.055 |
| 35 | WBPH | IR22 | 1 | 3 | 3.00 | 117.00 | 1.00 |  | 0.041 |
| 35 | WBPH | IR22 | 1 | 4 | 4.00 | 0.00 | 0.00 |  | 0.046 |
| 35 | WBPH | IR22 | 1 | 5 | 5.00 | 51.50 | 0.00 |  | 0.056 |
| 35 | WBPH | IR22 | 2 | 1 | 1.00 | 86.00 | 1.00 |  | 0.041 |
| 35 | WBPH | IR22 | 2 | 2 | 2.00 | 89.00 | 1.00 |  | 0.050 |
| 35 | WBPH | IR22 | 2 | 3 | 3.00 | 172.00 | 2.00 |  | 0.049 |
| 35 | WBPH | IR22 | 2 | 4 | 4.00 | 6.00 | 0.00 |  | 0.065 |
| 35 | WBPH | IR22 | 2 | 5 | 5.00 | 118.00 | 1.00 |  | 0.054 |
| 35 | WBPH | IR22 | 4 | 1 | 1.00 | 232.00 | 3.00 |  | 0.040 |
| 35 | WBPH | IR22 | 4 | 2 | 2.00 | 275.00 | 3.00 |  | 0.047 |
| 35 | WBPH | IR22 | 4 | 3 | 3.00 | 99.00 | 2.00 |  | 0.062 |
| 35 | WBPH | IR22 | 4 | 4 | 4.00 | 228.00 | 3.00 |  | 0.055 |
| 35 | WBPH | IR22 | 4 | 5 | 5.00 | 227.00 | 3.50 |  | 0.055 |
| 35 | WBPH | IR22 | 6 | 1 | 1.00 | 197.00 | 4.00 |  | 0.047 |
| 35 | WBPH | IR22 | 6 | 2 | 2.00 | 171.00 | 4.00 |  | 0.050 |
| 35 | WBPH | IR22 | 6 | 3 | 3.00 | 361.00 | 4.00 |  | 0.088 |
| 35 | WBPH | IR22 | 6 | 4 | 4.00 | 201.00 | 5.00 |  | 0.052 |
| 35 | WBPH | IR22 | 6 | 5 | 5.00 | 266.00 | 3.50 |  | 0.056 |
| 35 | WBPH | IR22 | 8 | 1 | 1.00 | 364.00 | 3.00 |  | 0.048 |
| 35 | WBPH | IR22 | 8 | 2 | 2.00 | 273.00 | 5.00 |  | 0.041 |
| 35 | WBPH | IR22 | 8 | 3 | 3.00 | 332.00 | 5.00 |  | 0.036 |
| 35 | WBPH | IR22 | 8 | 4 | 4.00 | 290.00 | 3.00 |  | 0.059 |
| 35 | WBPH | IR22 | 8 | 5 | 5.00 | 371.50 | 4.00 |  | 0.050 |
| 35 | WBPH | IR22 | 10 | 1 | 1.00 | 427.00 | 5.00 |  | 0.050 |
| 35 | WBPH | IR22 | 10 | 2 | 2.00 | 459.00 | 8.00 |  | 0.050 |
| 35 | WBPH | IR22 | 10 | 3 | 3.00 | 390.00 | 5.00 |  | 0.041 |
| 35 | WBPH | IR22 | 10 | 4 | 4.00 | 286.00 | 7.00 |  | 0.059 |
| 35 | WBPH | IR22 | 10 | 5 | 5.00 | 375.50 | 7.50 |  | 0.057 |
| 35 | WBPH | IR22 | 12 | 1 | 1.00 | 419.00 | 11.00 |  | 0.053 |
| 35 | WBPH | IR22 | 12 | 2 | 2.00 | 547.00 | 11.00 |  | 0.055 |
| 35 | WBPH | IR22 | 12 | 3 | 3.00 | 487.00 | 10.00 |  | 0.051 |
| 35 | WBPH | IR22 | 12 | 4 | 4.00 | 549.00 | 9.00 |  | 0.060 |
| 35 | WBPH | IR22 | 12 | 5 | 5.00 | 416.50 | 7.00 |  | 0.053 |
| 35 | WBPH | T65 | 1 | 1 | 1.00 | 68.00 | 0.00 |  | 0.064 |
| 35 | WBPH | T65 | 1 | 2 | 2.00 | 63.00 | 1.00 |  | 0.055 |
| 35 | WBPH | T65 | 1 | 3 | 3.00 | 51.00 | 0.00 |  | 0.064 |
| 35 | WBPH | T65 | 1 | 4 | 4.00 | 75.00 | 1.00 |  | 0.064 |
| 35 | WBPH | T65 | 1 | 5 | 5.00 | 79.00 | 1.00 |  | 0.056 |
| 35 | WBPH | T65 | 2 | 1 | 1.00 | 135.00 | 1.00 |  | 0.040 |
| 35 | WBPH | T65 | 2 | 2 | 2.00 | 183.00 | 1.00 |  | 0.036 |
| 35 | WBPH | T65 | 2 | 3 | 3.00 | 126.00 | 1.00 |  | 0.044 |
| 35 | WBPH | T65 | 2 | 4 | 4.00 | 198.00 | 2.00 |  | 0.054 |
| 35 | WBPH | T65 | 2 | 5 | 5.00 | 69.00 | 1.00 |  | 0.060 |
| 35 | WBPH | T65 | 4 | 1 | 1.00 | 203.00 | 2.00 |  | 0.035 |
| 35 | WBPH | T65 | 4 | 2 | 2.00 | 116.00 | 1.00 |  | 0.039 |
| 35 | WBPH | T65 | 4 | 3 | 3.00 | 177.00 | 3.00 |  | 0.044 |
| 35 | WBPH | T65 | 4 | 4 | 4.00 | 186.00 | 3.00 |  | 0.049 |
| 35 | WBPH | T65 | 4 | 5 | 5.00 | 184.00 | 4.00 |  | 0.031 |
| 35 | WBPH | T65 | 6 | 1 | 1.00 | 325.00 | 3.00 |  | 0.034 |
| 35 | WBPH | T65 | 6 | 2 | 2.00 | 247.00 | 3.00 |  | 0.053 |
| 35 | WBPH | T65 | 6 | 3 | 3.00 | 334.00 | 3.00 |  | 0.033 |
| 35 | WBPH | T65 | 6 | 4 | 4.00 | 300.00 | 5.00 |  | 0.038 |
| 35 | WBPH | T65 | 6 | 5 | 5.00 | 231.50 | 4.00 |  | 0.033 |
| 35 | WBPH | T65 | 8 | 1 | 1.00 | 421.00 | 5.00 |  | 0.042 |
| 35 | WBPH | T65 | 8 | 2 | 2.00 | 337.00 | 8.00 |  | 0.041 |
| 35 | WBPH | T65 | 8 | 3 | 3.00 | 374.00 | 6.00 |  | 0.050 |
| 35 | WBPH | T65 | 8 | 4 | 4.00 | 403.00 | 6.00 |  | 0.037 |
| 35 | WBPH | T65 | 8 | 5 | 5.00 | 373.00 | 6.00 |  | 0.052 |
| 35 | WBPH | T65 | 10 | 1 | 1.00 | 271.00 | 8.00 |  | 0.039 |
| 35 | WBPH | T65 | 10 | 2 | 2.00 | 401.00 | 8.00 |  | 0.048 |
| 35 | WBPH | T65 | 10 | 3 | 3.00 | 415.00 | 9.00 |  | 0.054 |
| 35 | WBPH | T65 | 10 | 4 | 4.00 | 311.00 | 6.00 |  | 0.033 |
| 35 | WBPH | T65 | 10 | 5 | 5.00 | 392.50 | 8.00 |  | 0.044 |
| 35 | WBPH | T65 | 12 | 1 | 1.00 | 437.00 | 11.00 |  | 0.033 |
| 35 | WBPH | T65 | 12 | 2 | 2.00 | 494.00 | 7.00 |  | 0.054 |
| 35 | WBPH | T65 | 12 | 3 | 3.00 | 465.00 | 8.00 |  | 0.055 |
| 35 | WBPH | T65 | 12 | 4 | 4.00 | 446.00 | 10.00 |  | 0.057 |
| 35 | WBPH | T65 | 12 | 5 | 5.00 | 424.00 | 10.50 |  | 0.054 |
| 35 | Control | IR22 | 0 | 1 |  |  |  |  | 0.044 |
| 35 | Control | IR22 | 0 | 3 |  |  |  |  | 0.064 |
| 35 | Control | IR22 | 0 | 4 |  |  |  |  | 0.046 |
| 30 | Control | IR22 | 0 | 1 |  |  |  |  | 0.042 |
| 30 | Control | IR22 | 0 | 3 |  |  |  |  | 0.053 |
| 30 | Control | IR22 | 0 | 4 |  |  |  |  | 0.039 |
| 25 | Control | IR22 | 0 | 1 |  |  |  |  | 0.040 |
| 25 | Control | IR22 | 0 | 3 |  |  |  |  | 0.047 |
| 25 | Control | IR22 | 0 | 4 |  |  |  |  | 0.047 |
| 35 | Control | T65 | 0 | 1 |  |  |  |  | 0.059 |
| 35 | Control | T65 | 0 | 3 |  |  |  |  | 0.035 |
| 35 | Control | T65 | 0 | 4 |  |  |  |  | 0.038 |
| 30 | Control | T65 | 0 | 1 |  |  |  |  | 0.038 |
| 30 | Control | T65 | 0 | 3 |  |  |  |  | 0.044 |
| 30 | Control | T65 | 0 | 4 |  |  |  |  | 0.060 |
| 25 | Control | T65 | 0 | 1 |  |  |  |  | 0.029 |
| 25 | Control | T65 | 0 | 3 |  |  |  |  | 0.045 |
| 25 | Control | T65 | 0 | 4 |  |  |  |  | 0.042 |
