# Supplementary material for: Intraspecific competition counters the effects of elevated and optimal temperatures on phloem-feeding insects in tropical and temperate rice
Source: PLoS One. 2020 Oct 6;15(10):e0240130. doi: 10.1371/journal.pone.0240130 (PMC7538200; doi:10.1371/journal.pone.0240130)
Supplement: S12 Table — (DOCX) [file pone.0240130.s012.docx]

**Table S12. Data from nymph experiments** (BPH = brown planthopper; WBPH = whitebacked planthopper; 1^st^ – 1^st^ instar, 2^nd^ = second instar, etc., Male = adult male survivor, Female = adult female survivor)

| Temperature (oC) | Species | Variety | Density | Run | 1st | 2nd | 3rd | 4th | 5th | Male | Female | adults | Planthopper dry weight (mg) | Plant biomass (g) |
| --- | --- | --- | --- | --- | --- | --- | --- | --- | --- | --- | --- | --- | --- | --- |
| 25 | BPH | IR22 | 5 | 1 | 0 | 0 | 0 | 5 | 0 | 0 | 0 | 0 | 1.35 | 0.053 |
| 25 | BPH | IR22 | 5 | 2 | 0 | 0 | 0 | 5 | 0 | 0 | 0 | 0 | 1.54 | 0.038 |
| 25 | BPH | IR22 | 5 | 3 | 0 | 0 | 0 | 3 | 2 | 0 | 0 | 0 | 1.37 | 0.056 |
| 25 | BPH | IR22 | 5 | 4 | 0 | 0 | 0 | 4 | 1 | 0 | 0 | 0 | 1.52 | 0.036 |
| 25 | BPH | IR22 | 5 | 5 | 0 | 0 | 0 | 4 | 1 | 0 | 0 | 0 | 1.48 | 0.051 |
| 25 | BPH | IR22 | 10 | 1 | 0 | 0 | 0 | 10 | 0 | 0 | 0 | 0 | 2.82 | 0.047 |
| 25 | BPH | IR22 | 10 | 2 | 0 | 0 | 0 | 10 | 0 | 0 | 0 | 0 | 2.75 | 0.042 |
| 25 | BPH | IR22 | 10 | 3 | 0 | 0 | 0 | 7 | 3 | 0 | 0 | 0 | 2.82 | 0.049 |
| 25 | BPH | IR22 | 10 | 4 | 0 | 0 | 0 | 6 | 2 | 0 | 1 | 1 | 3.04 | 0.049 |
| 25 | BPH | IR22 | 10 | 5 | 0 | 0 | 0 | 6 | 3 | 0 | 0 | 0 | 2.56 | 0.058 |
| 25 | BPH | IR22 | 15 | 1 | 0 | 0 | 0 | 14 | 0 | 0 | 0 | 0 | 3.60 | 0.049 |
| 25 | BPH | IR22 | 15 | 2 | 0 | 0 | 0 | 14 | 0 | 0 | 0 | 0 | 4.15 | 0.070 |
| 25 | BPH | IR22 | 15 | 3 | 0 | 0 | 0 | 4 | 11 | 0 | 0 | 0 | 4.62 | 0.061 |
| 25 | BPH | IR22 | 15 | 4 | 0 | 0 | 0 | 5 | 8 | 0 | 0 | 0 | 4.10 | 0.056 |
| 25 | BPH | IR22 | 15 | 5 | 0 | 0 | 0 | 6 | 9 | 0 | 0 | 0 | 4.72 | 0.053 |
| 25 | BPH | IR22 | 20 | 1 | 0 | 0 | 0 | 19 | 1 | 0 | 0 | 0 | 5.52 | 0.041 |
| 25 | BPH | IR22 | 20 | 2 | 0 | 0 | 0 | 17 | 2 | 0 | 0 | 0 | 4.72 | 0.047 |
| 25 | BPH | IR22 | 20 | 3 | 0 | 0 | 0 | 18 | 0 | 0 | 0 | 0 | 4.39 | 0.043 |
| 25 | BPH | IR22 | 20 | 4 | 0 | 0 | 0 | 18 | 0 | 0 | 0 | 0 | 4.42 | 0.070 |
| 25 | BPH | IR22 | 20 | 5 | 0 | 0 | 2 | 18 | 0 | 0 | 0 | 0 | 4.83 | 0.047 |
| 25 | BPH | IR22 | 25 | 1 | 0 | 0 | 0 | 24 | 0 | 0 | 0 | 0 | 6.31 | 0.043 |
| 25 | BPH | IR22 | 25 | 2 | 0 | 0 | 0 | 24 | 1 | 0 | 0 | 0 | 7.25 | 0.055 |
| 25 | BPH | IR22 | 25 | 3 | 0 | 0 | 0 | 24 | 1 | 0 | 0 | 0 | 7.24 | 0.041 |
| 25 | BPH | IR22 | 25 | 4 | 0 | 0 | 0 | 25 | 0 | 0 | 0 | 0 | 6.87 | 0.046 |
| 25 | BPH | IR22 | 25 | 5 | 0 | 0 | 0 | 25 | 0 | 0 | 0 | 0 | 6.42 | 0.056 |
| 25 | BPH | T65 | 5 | 1 | 0 | 0 | 0 | 5 | 0 | 0 | 0 | 0 | 1.38 | 0.041 |
| 25 | BPH | T65 | 5 | 2 | 0 | 0 | 0 | 5 | 0 | 0 | 0 | 0 | 1.24 | 0.050 |
| 25 | BPH | T65 | 5 | 3 | 0 | 0 | 0 | 3 | 2 | 0 | 0 | 0 | 1.65 | 0.049 |
| 25 | BPH | T65 | 5 | 4 | 0 | 0 | 0 | 4 | 1 | 0 | 0 | 0 | 1.52 | 0.065 |
| 25 | BPH | T65 | 5 | 5 | 0 | 0 | 0 | 2 | 3 | 0 | 0 | 0 | 1.58 | 0.054 |
| 25 | BPH | T65 | 10 | 1 | 0 | 0 | 0 | 10 | 0 | 0 | 0 | 0 | 2.83 | 0.040 |
| 25 | BPH | T65 | 10 | 2 | 0 | 0 | 0 | 9 | 0 | 0 | 0 | 0 | 2.30 | 0.047 |
| 25 | BPH | T65 | 10 | 3 | 0 | 0 | 0 | 2 | 7 | 0 | 0 | 0 | 2.72 | 0.062 |
| 25 | BPH | T65 | 10 | 4 | 0 | 0 | 0 | 4 | 6 | 0 | 0 | 0 | 3.24 | 0.055 |
| 25 | BPH | T65 | 10 | 5 | 0 | 0 | 0 | 3 | 6 | 0 | 0 | 0 | 2.94 | 0.055 |
| 25 | BPH | T65 | 15 | 1 | 0 | 0 | 0 | 12 | 0 | 0 | 0 | 0 | 2.98 | 0.047 |
| 25 | BPH | T65 | 15 | 2 | 0 | 0 | 0 | 15 | 0 | 0 | 0 | 0 | 3.71 | 0.050 |
| 25 | BPH | T65 | 15 | 3 | 0 | 0 | 0 | 11 | 3 | 0 | 0 | 0 | 4.31 | 0.088 |
| 25 | BPH | T65 | 15 | 4 | 0 | 0 | 0 | 11 | 4 | 0 | 0 | 0 | 4.24 | 0.052 |
| 25 | BPH | T65 | 15 | 5 | 0 | 0 | 0 | 10 | 4 | 0 | 0 | 0 | 3.93 | 0.056 |
| 25 | BPH | T65 | 20 | 1 | 0 | 0 | 0 | 20 | 0 | 0 | 0 | 0 | 5.26 | 0.048 |
| 25 | BPH | T65 | 20 | 2 | 0 | 0 | 0 | 20 | 0 | 0 | 0 | 0 | 5.40 | 0.041 |
| 25 | BPH | T65 | 20 | 3 | 0 | 0 | 0 | 19 | 0 | 0 | 0 | 0 | 4.91 | 0.036 |
| 25 | BPH | T65 | 20 | 4 | 0 | 0 | 0 | 19 | 0 | 0 | 0 | 0 | 5.03 | 0.059 |
| 25 | BPH | T65 | 20 | 5 | 0 | 0 | 0 | 17 | 0 | 0 | 0 | 0 | 4.32 | 0.050 |
| 25 | BPH | T65 | 25 | 1 | 0 | 0 | 0 | 23 | 0 | 0 | 0 | 0 | 6.36 | 0.050 |
| 25 | BPH | T65 | 25 | 2 | 0 | 0 | 0 | 23 | 0 | 0 | 0 | 0 | 6.13 | 0.050 |
| 25 | BPH | T65 | 25 | 3 | 0 | 0 | 1 | 23 | 0 | 0 | 0 | 0 | 6.53 | 0.041 |
| 25 | BPH | T65 | 25 | 4 | 0 | 0 | 0 | 22 | 0 | 0 | 0 | 0 | 5.04 | 0.059 |
| 25 | BPH | T65 | 25 | 5 | 0 | 0 | 0 | 25 | 0 | 0 | 0 | 0 | 6.70 | 0.057 |
| 25 | Control | IR22 | 0 | 1 |  |  |  |  |  |  |  |  |  | 0.053 |
| 25 | Control | IR22 | 0 | 2 |  |  |  |  |  |  |  |  |  | 0.055 |
| 25 | Control | IR22 | 0 | 3 |  |  |  |  |  |  |  |  |  | 0.051 |
| 25 | Control | IR22 | 0 | 4 |  |  |  |  |  |  |  |  |  | 0.060 |
| 25 | Control | IR22 | 0 | 5 |  |  |  |  |  |  |  |  |  | 0.053 |
| 25 | Control | T65 | 0 | 1 |  |  |  |  |  |  |  |  |  | 0.064 |
| 25 | Control | T65 | 0 | 2 |  |  |  |  |  |  |  |  |  | 0.055 |
| 25 | Control | T65 | 0 | 3 |  |  |  |  |  |  |  |  |  | 0.064 |
| 25 | Control | T65 | 0 | 4 |  |  |  |  |  |  |  |  |  | 0.064 |
| 25 | Control | T65 | 0 | 5 |  |  |  |  |  |  |  |  |  | 0.056 |
| 25 | WBPH | IR22 | 5 | 1 | 0 | 0 | 0 | 0 | 5 | 0 | 0 | 0 | 1.30 | 0.040 |
| 25 | WBPH | IR22 | 5 | 2 | 0 | 0 | 0 | 2 | 2 | 0 | 0 | 0 | 1.12 | 0.036 |
| 25 | WBPH | IR22 | 5 | 3 | 0 | 0 | 0 | 0 | 4 | 0 | 1 | 1 | 1.53 | 0.044 |
| 25 | WBPH | IR22 | 5 | 4 | 0 | 0 | 0 | 0 | 4 | 0 | 1 | 1 | 3.07 | 0.054 |
| 25 | WBPH | IR22 | 5 | 5 | 0 | 0 | 0 | 0 | 5 | 0 | 0 | 0 | 2.43 | 0.060 |
| 25 | WBPH | IR22 | 10 | 1 | 0 | 0 | 0 | 3 | 7 | 0 | 0 | 0 | 2.44 | 0.035 |
| 25 | WBPH | IR22 | 10 | 2 | 0 | 0 | 0 | 3 | 7 | 0 | 0 | 0 | 2.85 | 0.039 |
| 25 | WBPH | IR22 | 10 | 3 | 0 | 0 | 0 | 1 | 9 | 0 | 0 | 0 | 2.87 | 0.044 |
| 25 | WBPH | IR22 | 10 | 4 | 0 | 0 | 0 | 0 | 6 | 1 | 0 | 1 | 3.76 | 0.049 |
| 25 | WBPH | IR22 | 10 | 5 | 0 | 0 | 0 | 1 | 7 | 0 | 0 | 0 | 2.50 | 0.031 |
| 25 | WBPH | IR22 | 15 | 1 | 0 | 0 | 0 | 6 | 9 | 0 | 0 | 0 | 3.37 | 0.034 |
| 25 | WBPH | IR22 | 15 | 2 | 0 | 0 | 0 | 1 | 11 | 0 | 0 | 0 | 3.60 | 0.053 |
| 25 | WBPH | IR22 | 15 | 3 | 0 | 0 | 0 | 0 | 11 | 1 | 0 | 1 | 4.25 | 0.033 |
| 25 | WBPH | IR22 | 15 | 4 | 0 | 0 | 0 | 0 | 11 | 1 | 1 | 2 | 4.01 | 0.038 |
| 25 | WBPH | IR22 | 15 | 5 | 0 | 0 | 0 | 1 | 12 | 0 | 0 | 0 | 3.53 | 0.033 |
| 25 | WBPH | IR22 | 20 | 1 | 0 | 0 | 0 | 4 | 16 | 0 | 0 | 0 | 4.82 | 0.042 |
| 25 | WBPH | IR22 | 20 | 2 | 0 | 0 | 0 | 2 | 18 | 0 | 0 | 0 | 5.25 | 0.041 |
| 25 | WBPH | IR22 | 20 | 3 | 0 | 0 | 0 | 1 | 19 | 0 | 0 | 0 | 6.52 | 0.050 |
| 25 | WBPH | IR22 | 20 | 4 | 0 | 0 | 0 | 2 | 18 | 0 | 0 | 0 | 5.14 | 0.037 |
| 25 | WBPH | IR22 | 20 | 5 | 0 | 0 | 0 | 3 | 16 | 0 | 0 | 0 | 5.57 | 0.052 |
| 25 | WBPH | IR22 | 25 | 1 | 0 | 0 | 0 | 3 | 18 | 0 | 0 | 0 | 4.69 | 0.039 |
| 25 | WBPH | IR22 | 25 | 2 | 0 | 0 | 0 | 2 | 21 | 0 | 0 | 0 | 6.08 | 0.048 |
| 25 | WBPH | IR22 | 25 | 3 | 0 | 0 | 0 | 1 | 20 | 0 | 0 | 0 | 6.21 | 0.054 |
| 25 | WBPH | IR22 | 25 | 4 | 0 | 0 | 0 | 4 | 20 | 0 | 0 | 0 | 4.48 | 0.033 |
| 25 | WBPH | IR22 | 25 | 5 | 0 | 0 | 0 | 1 | 22 | 0 | 0 | 0 | 6.26 | 0.044 |
| 25 | WBPH | T65 | 5 | 1 | 0 | 0 | 0 | 2 | 3 | 0 | 0 | 0 | 1.09 | 0.033 |
| 25 | WBPH | T65 | 5 | 2 | 0 | 0 | 0 | 0 | 5 | 0 | 0 | 0 | 1.69 | 0.054 |
| 25 | WBPH | T65 | 5 | 3 | 0 | 0 | 0 | 1 | 4 | 0 | 0 | 0 | 1.77 | 0.055 |
| 25 | WBPH | T65 | 5 | 4 | 0 | 0 | 0 | 0 | 4 | 1 | 0 | 1 | 2.11 | 0.057 |
| 25 | WBPH | T65 | 5 | 5 | 0 | 0 | 0 | 0 | 4 | 1 | 0 | 1 | 2.04 | 0.054 |
| 25 | WBPH | T65 | 10 | 1 | 0 | 0 | 0 | 0 | 8 | 0 | 0 | 0 | 2.62 | 0.044 |
| 25 | WBPH | T65 | 10 | 2 | 0 | 0 | 0 | 1 | 9 | 0 | 0 | 0 | 2.80 | 0.064 |
| 25 | WBPH | T65 | 10 | 3 | 0 | 0 | 0 | 1 | 9 | 0 | 0 | 0 | 3.77 | 0.046 |
| 25 | WBPH | T65 | 10 | 4 | 0 | 0 | 0 | 0 | 6 | 2 | 1 | 3 | 3.53 | 0.042 |
| 25 | WBPH | T65 | 10 | 5 | 0 | 0 | 0 | 3 | 6 | 0 | 0 | 0 | 2.26 | 0.053 |
| 25 | WBPH | T65 | 15 | 1 | 0 | 0 | 0 | 3 | 12 | 0 | 0 | 0 | 4.24 | 0.039 |
| 25 | WBPH | T65 | 15 | 2 | 0 | 0 | 0 | 0 | 14 | 0 | 0 | 0 | 4.09 | 0.040 |
| 25 | WBPH | T65 | 15 | 3 | 0 | 0 | 0 | 0 | 10 | 4 | 0 | 4 | 6.33 | 0.047 |
| 25 | WBPH | T65 | 15 | 4 | 0 | 0 | 0 | 0 | 14 | 1 | 0 | 1 | 7.75 | 0.047 |
| 25 | WBPH | T65 | 15 | 5 | 0 | 0 | 0 | 0 | 12 | 3 | 0 | 3 | 6.73 | 0.059 |
| 25 | WBPH | T65 | 20 | 1 | 0 | 0 | 0 | 2 | 17 | 0 | 0 | 0 | 6.92 | 0.035 |
| 25 | WBPH | T65 | 20 | 2 | 0 | 0 | 0 | 1 | 19 | 0 | 0 | 0 | 5.21 | 0.038 |
| 25 | WBPH | T65 | 20 | 3 | 0 | 0 | 0 | 1 | 19 | 0 | 0 | 0 | 5.83 | 0.038 |
| 25 | WBPH | T65 | 20 | 4 | 0 | 0 | 0 | 2 | 17 | 0 | 0 | 0 | 6.75 | 0.044 |
| 25 | WBPH | T65 | 20 | 5 | 0 | 0 | 0 | 5 | 14 | 0 | 0 | 0 | 5.75 | 0.060 |
| 25 | WBPH | T65 | 25 | 1 | 0 | 0 | 0 | 1 | 22 | 0 | 0 | 0 | 6.16 | 0.029 |
| 25 | WBPH | T65 | 25 | 2 | 0 | 0 | 0 | 0 | 25 | 0 | 0 | 0 | 9.09 | 0.045 |
| 25 | WBPH | T65 | 25 | 3 | 0 | 0 | 0 | 3 | 19 | 0 | 0 | 0 | 6.21 | 0.042 |
| 25 | WBPH | T65 | 25 | 4 | 0 | 0 | 0 | 3 | 20 | 0 | 0 | 0 | 4.59 | 0.040 |
| 25 | WBPH | T65 | 25 | 5 | 0 | 0 | 0 | 2 | 23 | 0 | 0 | 0 | 7.37 | 0.061 |
| 30 | BPH | IR22 | 5 | 1 | 0 | 0 | 0 | 4 | 1 | 0 | 0 | 0 | 1.21 | 0.038 |
| 30 | BPH | IR22 | 5 | 2 | 0 | 0 | 0 | 1 | 4 | 0 | 0 | 0 | 1.21 | 0.045 |
| 30 | BPH | IR22 | 5 | 3 | 0 | 0 | 0 | 1 | 3 | 0 | 0 | 0 | 0.92 | 0.044 |
| 30 | BPH | IR22 | 5 | 4 | 0 | 0 | 0 | 1 | 4 | 0 | 0 | 0 | 1.38 | 0.045 |
| 30 | BPH | IR22 | 5 | 5 | 0 | 0 | 0 | 0 | 5 | 0 | 0 | 0 | 2.39 | 0.059 |
| 30 | BPH | IR22 | 10 | 1 | 0 | 0 | 1 | 8 | 1 | 0 | 0 | 0 | 1.77 | 0.051 |
| 30 | BPH | IR22 | 10 | 2 | 0 | 0 | 0 | 3 | 6 | 0 | 0 | 0 | 1.95 | 0.064 |
| 30 | BPH | IR22 | 10 | 3 | 0 | 0 | 0 | 1 | 9 | 0 | 0 | 0 | 3.48 | 0.056 |
| 30 | BPH | IR22 | 10 | 4 | 0 | 0 | 0 | 1 | 8 | 0 | 0 | 0 | 3.25 | 0.054 |
| 30 | BPH | IR22 | 10 | 5 | 0 | 0 | 0 | 1 | 8 | 0 | 0 | 0 | 3.71 | 0.056 |
| 30 | BPH | IR22 | 15 | 1 | 0 | 0 | 0 | 13 | 2 | 0 | 0 | 0 | 2.86 | 0.044 |
| 30 | BPH | IR22 | 15 | 2 | 0 | 0 | 0 | 8 | 5 | 0 | 0 | 0 | 3.04 | 0.056 |
| 30 | BPH | IR22 | 15 | 3 | 0 | 0 | 0 | 2 | 13 | 0 | 0 | 0 | 5.54 | 0.042 |
| 30 | BPH | IR22 | 15 | 4 | 0 | 0 | 0 | 2 | 12 | 0 | 0 | 0 | 4.49 | 0.040 |
| 30 | BPH | IR22 | 15 | 5 | 0 | 0 | 0 | 3 | 12 | 0 | 0 | 0 | 4.57 | 0.034 |
| 30 | BPH | IR22 | 20 | 1 | 0 | 0 | 0 | 13 | 7 | 0 | 0 | 0 | 4.11 | 0.050 |
| 30 | BPH | IR22 | 20 | 2 | 0 | 0 | 0 | 9 | 10 | 0 | 0 | 0 | 4.52 | 0.041 |
| 30 | BPH | IR22 | 20 | 3 | 0 | 0 | 0 | 12 | 7 | 0 | 0 | 0 | 3.93 | 0.055 |
| 30 | BPH | IR22 | 20 | 4 | 0 | 0 | 2 | 12 | 5 | 0 | 0 | 0 | 3.04 | 0.051 |
| 30 | BPH | IR22 | 20 | 5 | 0 | 0 | 1 | 15 | 3 | 0 | 0 | 0 | 3.42 | 0.049 |
| 30 | BPH | IR22 | 25 | 1 | 0 | 0 | 0 | 14 | 10 | 0 | 0 | 0 | 4.92 | 0.042 |
| 30 | BPH | IR22 | 25 | 2 | 0 | 0 | 1 | 15 | 8 | 0 | 0 | 0 | 5.09 | 0.051 |
| 30 | BPH | IR22 | 25 | 3 | 0 | 0 | 1 | 18 | 4 | 0 | 0 | 0 | 5.25 | 0.048 |
| 30 | BPH | IR22 | 25 | 4 | 0 | 0 | 2 | 22 | 0 | 0 | 0 | 0 | 4.08 | 0.041 |
| 30 | BPH | IR22 | 25 | 5 | 0 | 0 | 7 | 18 | 0 | 0 | 0 | 0 | 3.79 | 0.035 |
| 30 | BPH | T65 | 5 | 1 | 0 | 0 | 1 | 4 | 0 | 0 | 0 | 0 | 0.75 | 0.049 |
| 30 | BPH | T65 | 5 | 2 | 0 | 0 | 1 | 3 | 0 | 0 | 0 | 0 | 0.54 | 0.039 |
| 30 | BPH | T65 | 5 | 3 | 0 | 0 | 0 | 1 | 3 | 0 | 0 | 0 | 1.49 | 0.074 |
| 30 | BPH | T65 | 5 | 4 | 0 | 0 | 0 | 2 | 3 | 0 | 0 | 0 | 1.46 | 0.050 |
| 30 | BPH | T65 | 5 | 5 | 0 | 0 | 0 | 1 | 4 | 0 | 0 | 0 | 2.02 | 0.056 |
| 30 | BPH | T65 | 10 | 1 | 0 | 0 | 0 | 8 | 2 | 0 | 0 | 0 | 1.72 | 0.055 |
| 30 | BPH | T65 | 10 | 2 | 0 | 0 | 2 | 7 | 1 | 0 | 0 | 0 | 1.69 | 0.053 |
| 30 | BPH | T65 | 10 | 3 | 0 | 0 | 0 | 1 | 8 | 0 | 0 | 0 | 2.95 | 0.065 |
| 30 | BPH | T65 | 10 | 4 | 0 | 0 | 0 | 2 | 8 | 0 | 0 | 0 | 2.89 | 0.056 |
| 30 | BPH | T65 | 10 | 5 | 0 | 0 | 0 | 1 | 8 | 0 | 0 | 0 | 3.50 | 0.039 |
| 30 | BPH | T65 | 15 | 1 | 0 | 0 | 3 | 5 | 5 | 0 | 0 | 0 | 2.46 | 0.061 |
| 30 | BPH | T65 | 15 | 2 | 0 | 0 | 1 | 9 | 4 | 0 | 0 | 0 | 2.98 | 0.056 |
| 30 | BPH | T65 | 15 | 3 | 0 | 0 | 0 | 1 | 11 | 0 | 0 | 0 | 4.10 | 0.056 |
| 30 | BPH | T65 | 15 | 4 | 0 | 0 | 0 | 5 | 10 | 0 | 0 | 0 | 5.14 | 0.043 |
| 30 | BPH | T65 | 15 | 5 | 0 | 0 | 0 | 3 | 9 | 0 | 0 | 0 | 5.46 | 0.044 |
| 30 | BPH | T65 | 20 | 1 | 0 | 0 | 2 | 6 | 6 | 0 | 0 | 0 | 5.51 | 0.047 |
| 30 | BPH | T65 | 20 | 2 | 0 | 0 | 0 | 14 | 5 | 0 | 0 | 0 | 3.56 | 0.055 |
| 30 | BPH | T65 | 20 | 3 | 0 | 0 | 1 | 11 | 2 | 0 | 0 | 0 | 4.22 | 0.059 |
| 30 | BPH | T65 | 20 | 4 | 0 | 0 | 1 | 7 | 8 | 0 | 0 | 0 | 3.55 | 0.057 |
| 30 | BPH | T65 | 20 | 5 | 0 | 0 | 1 | 12 | 1 | 0 | 0 | 0 | 2.88 | 0.060 |
| 30 | BPH | T65 | 25 | 1 | 0 | 0 | 1 | 20 | 4 | 0 | 0 | 0 | 4.42 | 0.072 |
| 30 | BPH | T65 | 25 | 2 | 0 | 0 | 4 | 17 | 1 | 0 | 0 | 0 | 3.67 | 0.043 |
| 30 | BPH | T65 | 25 | 3 | 0 | 0 | 1 | 13 | 9 | 0 | 0 | 0 | 5.66 | 0.076 |
| 30 | BPH | T65 | 25 | 4 | 0 | 0 | 2 | 20 | 3 | 0 | 0 | 0 | 3.68 | 0.03 |
| 30 | BPH | T65 | 25 | 5 | 0 | 0 | 1 | 11 | 7 | 0 | 0 | 0 | 4.17 | 0.063 |
| 30 | Control | IR22 | 0 | 1 |  |  |  |  |  |  |  |  |  | 0.058 |
| 30 | Control | IR22 | 0 | 2 |  |  |  |  |  |  |  |  |  | 0.064 |
| 30 | Control | IR22 | 0 | 3 |  |  |  |  |  |  |  |  |  | 0.062 |
| 30 | Control | IR22 | 0 | 4 |  |  |  |  |  |  |  |  |  | 0.058 |
| 30 | Control | IR22 | 0 | 5 |  |  |  |  |  |  |  |  |  | 0.059 |
| 30 | Control | T65 | 0 | 1 |  |  |  |  |  |  |  |  |  | 0.055 |
| 30 | Control | T65 | 0 | 2 |  |  |  |  |  |  |  |  |  | 0.055 |
| 30 | Control | T65 | 0 | 3 |  |  |  |  |  |  |  |  |  | 0.078 |
| 30 | Control | T65 | 0 | 4 |  |  |  |  |  |  |  |  |  | 0.060 |
| 30 | Control | T65 | 0 | 5 |  |  |  |  |  |  |  |  |  | 0.074 |
| 30 | WBPH | IR22 | 5 | 1 | 0 | 0 | 0 | 3 | 2 | 0 | 0 | 0 | 0.57 | 0.049 |
| 30 | WBPH | IR22 | 5 | 2 | 0 | 0 | 0 | 4 | 1 | 0 | 0 | 0 | 0.52 | 0.037 |
| 30 | WBPH | IR22 | 5 | 3 | 0 | 0 | 0 | 0 | 5 | 0 | 0 | 0 | 1.23 | 0.049 |
| 30 | WBPH | IR22 | 5 | 4 | 0 | 0 | 0 | 0 | 4 | 0 | 0 | 0 | 0.63 | 0.046 |
| 30 | WBPH | IR22 | 5 | 5 | 0 | 0 | 0 | 1 | 3 | 0 | 0 | 0 | 0.53 | 0.058 |
| 30 | WBPH | IR22 | 10 | 1 | 0 | 0 | 0 | 2 | 6 | 0 | 0 | 0 | 1.23 | 0.044 |
| 30 | WBPH | IR22 | 10 | 2 | 0 | 0 | 1 | 5 | 2 | 0 | 0 | 0 | 0.99 | 0.042 |
| 30 | WBPH | IR22 | 10 | 3 | 0 | 0 | 0 | 3 | 7 | 0 | 0 | 0 | 1.80 | 0.051 |
| 30 | WBPH | IR22 | 10 | 4 | 0 | 0 | 0 | 1 | 4 | 0 | 1 | 1 | 1.42 | 0.047 |
| 30 | WBPH | IR22 | 10 | 5 | 0 | 0 | 0 | 2 | 7 | 1 | 0 | 1 | 1.32 | 0.040 |
| 30 | WBPH | IR22 | 15 | 1 | 0 | 0 | 0 | 2 | 9 | 0 | 0 | 0 | 1.59 | 0.047 |
| 30 | WBPH | IR22 | 15 | 2 | 0 | 0 | 0 | 2 | 9 | 0 | 0 | 0 | 1.61 | 0.057 |
| 30 | WBPH | IR22 | 15 | 3 | 0 | 0 | 0 | 0 | 13 | 0 | 0 | 0 | 2.99 | 0.043 |
| 30 | WBPH | IR22 | 15 | 4 | 0 | 0 | 0 | 6 | 7 | 0 | 0 | 0 | 1.65 | 0.041 |
| 30 | WBPH | IR22 | 15 | 5 | 0 | 0 | 0 | 0 | 7 | 0 | 0 | 0 | 1.02 | 0.052 |
| 30 | WBPH | IR22 | 20 | 1 | 0 | 0 | 5 | 12 | 1 | 0 | 0 | 0 | 1.26 | 0.042 |
| 30 | WBPH | IR22 | 20 | 2 | 0 | 0 | 0 | 7 | 9 | 0 | 0 | 0 | 2.17 | 0.048 |
| 30 | WBPH | IR22 | 20 | 3 | 0 | 0 | 1 | 13 | 4 | 0 | 0 | 0 | 2.12 | 0.048 |
| 30 | WBPH | IR22 | 20 | 4 | 0 | 0 | 3 | 11 | 6 | 0 | 0 | 0 | 2.05 | 0.034 |
| 30 | WBPH | IR22 | 20 | 5 | 0 | 0 | 2 | 7 | 9 | 1 | 0 | 1 | 2.23 | 0.039 |
| 30 | WBPH | IR22 | 25 | 1 | 0 | 0 | 2 | 16 | 7 | 0 | 0 | 0 | 2.37 | 0.04 |
| 30 | WBPH | IR22 | 25 | 2 | 0 | 0 | 0 | 14 | 10 | 0 | 0 | 0 | 2.62 | 0.047 |
| 30 | WBPH | IR22 | 25 | 3 | 0 | 0 | 0 | 5 | 19 | 0 | 0 | 0 | 3.29 | 0.048 |
| 30 | WBPH | IR22 | 25 | 4 | 0 | 0 | 0 | 5 | 12 | 1 | 0 | 1 | 3.57 | 0.052 |
| 30 | WBPH | IR22 | 25 | 5 | 0 | 0 | 1 | 17 | 3 | 0 | 0 | 0 | 1.97 | 0.034 |
| 30 | WBPH | T65 | 5 | 1 | 0 | 0 | 0 | 1 | 2 | 0 | 0 | 0 | 0.29 | 0.06 |
| 30 | WBPH | T65 | 5 | 2 | 0 | 0 | 0 | 4 | 0 | 0 | 0 | 0 | 0.43 | 0.05 |
| 30 | WBPH | T65 | 5 | 3 | 0 | 0 | 0 | 1 | 3 | 0 | 0 | 0 | 0.63 | 0.054 |
| 30 | WBPH | T65 | 5 | 4 | 0 | 0 | 0 | 0 | 3 | 0 | 0 | 0 | 0.60 | 0.057 |
| 30 | WBPH | T65 | 5 | 5 | 0 | 0 | 0 | 3 | 2 | 0 | 0 | 0 | 0.65 | 0.067 |
| 30 | WBPH | T65 | 10 | 1 | 0 | 0 | 0 | 3 | 3 | 0 | 0 | 0 | 0.67 | 0.054 |
| 30 | WBPH | T65 | 10 | 2 | 0 | 0 | 0 | 3 | 4 | 0 | 0 | 0 | 0.86 | 0.062 |
| 30 | WBPH | T65 | 10 | 3 | 0 | 0 | 0 | 0 | 8 | 0 | 0 | 0 | 1.56 | 0.064 |
| 30 | WBPH | T65 | 10 | 4 | 0 | 0 | 0 | 1 | 6 | 1 | 0 | 1 | 1.46 | 0.07 |
| 30 | WBPH | T65 | 10 | 5 | 0 | 0 | 0 | 7 | 1 | 0 | 0 | 0 | 0.78 | 0.044 |
| 30 | WBPH | T65 | 15 | 1 | 0 | 0 | 1 | 0 | 10 | 0 | 0 | 0 | 1.54 | 0.05 |
| 30 | WBPH | T65 | 15 | 2 | 0 | 0 | 0 | 4 | 8 | 0 | 0 | 0 | 1.51 | 0.063 |
| 30 | WBPH | T65 | 15 | 3 | 0 | 0 | 0 | 8 | 4 | 0 | 0 | 0 | 1.15 | 0.062 |
| 30 | WBPH | T65 | 15 | 4 | 0 | 0 | 0 | 0 | 8 | 0 | 0 | 0 | 1.79 | 0.06 |
| 30 | WBPH | T65 | 15 | 5 | 0 | 0 | 0 | 7 | 3 | 0 | 0 | 0 | 1.17 | 0.06 |
| 30 | WBPH | T65 | 20 | 1 | 0 | 0 | 1 | 3 | 12 | 0 | 0 | 0 | 2.63 | 0.063 |
| 30 | WBPH | T65 | 20 | 2 | 0 | 0 | 1 | 5 | 10 | 0 | 0 | 0 | 2.25 | 0.05 |
| 30 | WBPH | T65 | 20 | 3 | 0 | 0 | 0 | 3 | 14 | 0 | 0 | 0 | 2.54 | 0.07 |
| 30 | WBPH | T65 | 20 | 4 | 0 | 0 | 1 | 8 | 10 | 0 | 0 | 0 | 2.20 | 0.042 |
| 30 | WBPH | T65 | 20 | 5 | 0 | 0 | 1 | 3 | 14 | 0 | 0 | 0 | 2.75 | 0.06 |
| 30 | WBPH | T65 | 25 | 1 | 0 | 0 | 0 | 2 | 19 | 1 | 0 | 1 | 3.93 | 0.055 |
| 30 | WBPH | T65 | 25 | 2 | 0 | 0 | 1 | 2 | 19 | 0 | 0 | 0 | 3.36 | 0.048 |
| 30 | WBPH | T65 | 25 | 3 | 0 | 0 | 1 | 5 | 17 | 0 | 0 | 0 | 3.08 | 0.063 |
| 30 | WBPH | T65 | 25 | 4 | 0 | 0 | 3 | 8 | 9 | 0 | 0 | 0 | 2.59 | 0.046 |
| 30 | WBPH | T65 | 25 | 5 | 0 | 0 | 0 | 2 | 22 | 1 | 0 | 1 | 3.68 | 0.061 |
| 35 | BPH | IR22 | 5 | 1 | 0 | 1 | 0 | 0 | 0 | 0 | 0 | 0 | 0.02 | 0.016 |
| 35 | BPH | IR22 | 5 | 2 | 0 | 4 | 0 | 0 | 0 | 0 | 0 | 0 | 0.05 | 0.011 |
| 35 | BPH | IR22 | 5 | 3 | 0 | 2 | 1 | 0 | 0 | 0 | 0 | 0 | 0.06 | 0.022 |
| 35 | BPH | IR22 | 5 | 4 | 0 | 0 | 1 | 0 | 0 | 0 | 0 | 0 | 0.05 | 0.015 |
| 35 | BPH | IR22 | 5 | 5 | 0 | 1 | 1 | 0 | 0 | 0 | 0 | 0 | 0.03 | 0.012 |
| 35 | BPH | IR22 | 10 | 1 | 0 | 0 | 6 | 0 | 0 | 0 | 0 | 0 | 0.18 | 0.013 |
| 35 | BPH | IR22 | 10 | 2 | 0 | 0 | 1 | 0 | 0 | 0 | 0 | 0 | 0.03 | 0.016 |
| 35 | BPH | IR22 | 10 | 3 | 0 | 0 | 4 | 0 | 0 | 0 | 0 | 0 | 0.15 | 0.016 |
| 35 | BPH | IR22 | 10 | 4 | 0 | 2 | 0 | 0 | 0 | 0 | 0 | 0 | 0.03 | 0.02 |
| 35 | BPH | IR22 | 10 | 5 | 0 | 1 | 1 | 0 | 0 | 0 | 0 | 0 | 0.04 | 0.019 |
| 35 | BPH | IR22 | 15 | 1 | 0 | 0 | 9 | 0 | 0 | 0 | 0 | 0 | 0.24 | 0.036 |
| 35 | BPH | IR22 | 15 | 2 | 0 | 2 | 4 | 0 | 0 | 0 | 0 | 0 | 0.08 | 0.019 |
| 35 | BPH | IR22 | 15 | 3 | 0 | 6 | 5 | 0 | 0 | 0 | 0 | 0 | 0.18 | 0.013 |
| 35 | BPH | IR22 | 15 | 4 | 0 | 1 | 8 | 0 | 0 | 0 | 0 | 0 | 0.17 | 0.022 |
| 35 | BPH | IR22 | 15 | 5 | 0 | 1 | 4 | 0 | 0 | 0 | 0 | 0 | 0.11 | 0.018 |
| 35 | BPH | IR22 | 20 | 1 | 0 | 3 | 6 | 0 | 0 | 0 | 0 | 0 | 0.17 | 0.018 |
| 35 | BPH | IR22 | 20 | 2 | 0 | 8 | 3 | 0 | 0 | 0 | 0 | 0 | 0.18 | 0.023 |
| 35 | BPH | IR22 | 20 | 3 | 0 | 2 | 9 | 0 | 0 | 0 | 0 | 0 | 0.19 | 0.022 |
| 35 | BPH | IR22 | 20 | 4 | 0 | 4 | 3 | 0 | 0 | 0 | 0 | 0 | 0.19 | 0.031 |
| 35 | BPH | IR22 | 20 | 5 | 0 | 3 | 7 | 0 | 0 | 0 | 0 | 0 | 0.22 | 0.013 |
| 35 | BPH | IR22 | 25 | 1 | 0 | 5 | 5 | 0 | 0 | 0 | 0 | 0 | 0.15 | 0.02 |
| 35 | BPH | IR22 | 25 | 2 | 0 | 6 | 3 | 0 | 1 | 0 | 0 | 0 | 0.68 | 0.021 |
| 35 | BPH | IR22 | 25 | 3 | 0 | 4 | 5 | 0 | 0 | 0 | 0 | 0 | 0.11 | 0.019 |
| 35 | BPH | IR22 | 25 | 4 | 0 | 3 | 7 | 0 | 0 | 0 | 0 | 0 | 0.20 | 0.024 |
| 35 | BPH | IR22 | 25 | 5 | 0 | 4 | 6 | 1 | 0 | 0 | 0 | 0 | 0.20 | 0.024 |
| 35 | BPH | T65 | 5 | 1 | 0 | 1 | 2 | 0 | 0 | 0 | 0 | 0 | 0.05 | 0.024 |
| 35 | BPH | T65 | 5 | 2 | 0 | 2 | 1 | 0 | 0 | 0 | 0 | 0 | 0.09 | 0.024 |
| 35 | BPH | T65 | 5 | 3 | 0 | 2 | 1 | 0 | 0 | 0 | 0 | 0 | 0.07 | 0.025 |
| 35 | BPH | T65 | 5 | 4 | 0 | 2 | 0 | 0 | 1 | 0 | 0 | 0 | 0.14 | 0.025 |
| 35 | BPH | T65 | 5 | 5 | 0 | 4 | 6 | 0 | 0 | 0 | 0 | 0 | 0.15 | 0.025 |
| 35 | BPH | T65 | 10 | 1 | 0 | 2 | 3 | 0 | 0 | 0 | 0 | 0 | 0.14 | 0.021 |
| 35 | BPH | T65 | 10 | 2 | 0 | 0 | 4 | 0 | 0 | 0 | 0 | 0 | 0.12 | 0.016 |
| 35 | BPH | T65 | 10 | 3 | 0 | 0 | 1 | 0 | 0 | 0 | 0 | 0 | 0.02 | 0.017 |
| 35 | BPH | T65 | 10 | 4 | 0 | 2 | 4 | 0 | 0 | 0 | 0 | 0 | 0.24 | 0.023 |
| 35 | BPH | T65 | 10 | 5 | 0 | 0 | 1 | 0 | 0 | 0 | 0 | 0 | 0.02 | 0.021 |
| 35 | BPH | T65 | 15 | 1 | 0 | 0 | 10 | 0 | 0 | 0 | 0 | 0 | 0.35 | 0.019 |
| 35 | BPH | T65 | 15 | 2 | 0 | 0 | 5 | 0 | 0 | 0 | 0 | 0 | 0.12 | 0.019 |
| 35 | BPH | T65 | 15 | 3 | 0 | 3 | 6 | 0 | 0 | 0 | 0 | 0 | 0.21 | 0.019 |
| 35 | BPH | T65 | 15 | 4 | 0 | 2 | 5 | 0 | 0 | 0 | 0 | 0 | 0.15 | 0.018 |
| 35 | BPH | T65 | 15 | 5 | 0 | 0 | 4 | 1 | 0 | 0 | 0 | 0 | 0.16 | 0.014 |
| 35 | BPH | T65 | 20 | 1 | 0 | 4 | 11 | 0 | 0 | 0 | 0 | 0 | 0.48 | 0.019 |
| 35 | BPH | T65 | 20 | 2 | 0 | 3 | 5 | 0 | 0 | 0 | 0 | 0 | 0.26 | 0.012 |
| 35 | BPH | T65 | 20 | 3 | 0 | 2 | 9 | 0 | 0 | 0 | 0 | 0 | 0.25 | 0.021 |
| 35 | BPH | T65 | 20 | 4 | 0 | 0 | 6 | 0 | 0 | 0 | 0 | 0 | 0.19 | 0.021 |
| 35 | BPH | T65 | 20 | 5 | 0 | 0 | 0 | 0 | 0 | 0 | 0 | 0 | 0.29 | 0.02 |
| 35 | BPH | T65 | 25 | 1 | 0 | 1 | 7 | 0 | 0 | 0 | 0 | 0 | 0.25 | 0.017 |
| 35 | BPH | T65 | 25 | 2 | 0 | 1 | 10 | 0 | 1 | 0 | 0 | 0 | 0.45 | 0.023 |
| 35 | BPH | T65 | 25 | 3 | 0 | 1 | 4 | 0 | 0 | 0 | 0 | 0 | 0.13 | 0.025 |
| 35 | BPH | T65 | 25 | 4 | 0 | 0 | 4 | 0 | 0 | 0 | 0 | 0 | 0.12 | 0.024 |
| 35 | BPH | T65 | 25 | 5 | 0 | 0 | 8 | 0 | 0 | 0 | 0 | 0 | 0.11 | 0.02 |
| 35 | Control | IR22 | 0 | 1 |  |  |  |  |  |  |  |  |  | 0.022 |
| 35 | Control | IR22 | 0 | 2 |  |  |  |  |  |  |  |  |  | 0.022 |
| 35 | Control | IR22 | 0 | 3 |  |  |  |  |  |  |  |  |  | 0.023 |
| 35 | Control | IR22 | 0 | 4 |  |  |  |  |  |  |  |  |  | 0.025 |
| 35 | Control | IR22 | 0 | 5 |  |  |  |  |  |  |  |  |  | 0.02 |
| 35 | Control | T65 | 0 | 1 |  |  |  |  |  |  |  |  |  | 0.024 |
| 35 | Control | T65 | 0 | 2 |  |  |  |  |  |  |  |  |  | 0.025 |
| 35 | Control | T65 | 0 | 3 |  |  |  |  |  |  |  |  |  | 0.024 |
| 35 | Control | T65 | 0 | 4 |  |  |  |  |  |  |  |  |  | 0.025 |
| 35 | Control | T65 | 0 | 5 |  |  |  |  |  |  |  |  |  | 0.025 |
| 35 | WBPH | IR22 | 5 | 1 | 0 | 0 | 2 | 0 | 0 | 0 | 0 | 0 | 0.04 | 0.015 |
| 35 | WBPH | IR22 | 5 | 2 | 0 | 0 | 1 | 1 | 0 | 0 | 0 | 0 | 0.04 | 0.015 |
| 35 | WBPH | IR22 | 5 | 3 | 0 | 1 | 2 | 0 | 0 | 0 | 0 | 0 | 0.05 | 0.013 |
| 35 | WBPH | IR22 | 5 | 4 | 0 | 0 | 2 | 0 | 0 | 0 | 0 | 0 | 0.03 | 0.011 |
| 35 | WBPH | IR22 | 5 | 5 | 0 | 0 | 1 | 0 | 0 | 0 | 0 | 0 | 0.03 | 0.02 |
| 35 | WBPH | IR22 | 10 | 1 | 0 | 0 | 1 | 0 | 0 | 0 | 0 | 0 | 0.01 | 0.015 |
| 35 | WBPH | IR22 | 10 | 2 | 0 | 0 | 5 | 0 | 0 | 0 | 0 | 0 | 0.09 | 0.02 |
| 35 | WBPH | IR22 | 10 | 3 | 0 | 0 | 2 | 2 | 0 | 0 | 0 | 0 | 0.09 | 0.01 |
| 35 | WBPH | IR22 | 10 | 4 | 0 | 0 | 0 | 1 | 0 | 0 | 0 | 0 | 0.04 | 0.012 |
| 35 | WBPH | IR22 | 10 | 5 | 0 | 1 | 1 | 0 | 0 | 0 | 0 | 0 | 0.03 | 0.017 |
| 35 | WBPH | IR22 | 15 | 1 | 0 | 0 | 0 | 3 | 0 | 0 | 0 | 0 | 0.06 | 0.014 |
| 35 | WBPH | IR22 | 15 | 2 | 0 | 0 | 2 | 1 | 0 | 0 | 0 | 0 | 0.04 | 0.019 |
| 35 | WBPH | IR22 | 15 | 3 | 0 | 0 | 0 | 0 | 0 | 0 | 0 | 0 | 0.001* | 0.012 |
| 35 | WBPH | IR22 | 15 | 4 | 0 | 0 | 0 | 0 | 0 | 0 | 0 | 0 | 0.001* | 0.017 |
| 35 | WBPH | IR22 | 15 | 5 | 0 | 0 | 1 | 2 | 0 | 0 | 0 | 0 | 0.04 | 0.01 |
| 35 | WBPH | IR22 | 20 | 1 | 0 | 0 | 0 | 0 | 0 | 0 | 0 | 0 | 0.001* | 0.024 |
| 35 | WBPH | IR22 | 20 | 2 | 0 | 0 | 0 | 0 | 0 | 0 | 0 | 0 | 0.001* | 0.015 |
| 35 | WBPH | IR22 | 20 | 3 | 0 | 0 | 2 | 4 | 0 | 0 | 0 | 0 | 0.12 | 0.02 |
| 35 | WBPH | IR22 | 20 | 4 | 0 | 0 | 1 | 3 | 0 | 0 | 0 | 0 | 0.08 | 0.011 |
| 35 | WBPH | IR22 | 20 | 5 | 0 | 1 | 1 | 0 | 0 | 0 | 0 | 0 | 0.03 | 0.018 |
| 35 | WBPH | IR22 | 25 | 1 | 0 | 1 | 7 | 0 | 0 | 0 | 0 | 0 | 0.16 | 0.012 |
| 35 | WBPH | IR22 | 25 | 2 | 0 | 0 | 3 | 0 | 0 | 0 | 0 | 0 | 0.07 | 0.022 |
| 35 | WBPH | IR22 | 25 | 3 | 0 | 0 | 12 | 5 | 0 | 0 | 0 | 0 | 0.38 | 0.012 |
| 35 | WBPH | IR22 | 25 | 4 | 0 | 1 | 7 | 7 | 0 | 0 | 0 | 0 | 0.35 | 0.02 |
| 35 | WBPH | IR22 | 25 | 5 | 0 | 1 | 6 | 0 | 0 | 0 | 0 | 0 | 0.11 | 0.025 |
| 35 | WBPH | T65 | 5 | 1 | 0 | 0 | 2 | 0 | 0 | 0 | 0 | 0 | 0.07 | 0.019 |
| 35 | WBPH | T65 | 5 | 2 | 0 | 0 | 2 | 0 | 0 | 0 | 0 | 0 | 0.06 | 0.019 |
| 35 | WBPH | T65 | 5 | 3 | 0 | 0 | 1 | 0 | 0 | 0 | 0 | 0 | 0.02 | 0.018 |
| 35 | WBPH | T65 | 5 | 4 | 0 | 0 | 2 | 0 | 0 | 0 | 0 | 0 | 0.04 | 0.021 |
| 35 | WBPH | T65 | 5 | 5 | 0 | 0 | 2 | 1 | 0 | 0 | 0 | 0 | 0.07 | 0.025 |
| 35 | WBPH | T65 | 10 | 1 | 0 | 0 | 4 | 3 | 0 | 0 | 0 | 0 | 0.20 | 0.02 |
| 35 | WBPH | T65 | 10 | 2 | 0 | 0 | 5 | 0 | 0 | 0 | 0 | 0 | 0.15 | 0.014 |
| 35 | WBPH | T65 | 10 | 3 | 0 | 1 | 3 | 0 | 0 | 0 | 0 | 0 | 0.09 | 0.016 |
| 35 | WBPH | T65 | 10 | 4 | 0 | 0 | 3 | 1 | 0 | 0 | 0 | 0 | 0.15 | 0.018 |
| 35 | WBPH | T65 | 10 | 5 | 0 | 0 | 3 | 0 | 0 | 0 | 0 | 0 | 0.08 | 0.021 |
| 35 | WBPH | T65 | 15 | 1 | 0 | 1 | 5 | 0 | 0 | 0 | 0 | 0 | 0.16 | 0.021 |
| 35 | WBPH | T65 | 15 | 2 | 0 | 0 | 5 | 0 | 0 | 0 | 0 | 0 | 0.17 | 0.026 |
| 35 | WBPH | T65 | 15 | 3 | 0 | 2 | 6 | 1 | 0 | 0 | 0 | 0 | 0.22 | 0.016 |
| 35 | WBPH | T65 | 15 | 4 | 0 | 1 | 2 | 2 | 0 | 0 | 0 | 0 | 0.11 | 0.019 |
| 35 | WBPH | T65 | 15 | 5 | 0 | 1 | 4 | 0 | 0 | 0 | 0 | 0 | 0.14 | 0.018 |
| 35 | WBPH | T65 | 20 | 1 | 0 | 0 | 4 | 2 | 0 | 0 | 0 | 0 | 0.22 | 0.027 |
| 35 | WBPH | T65 | 20 | 2 | 0 | 0 | 5 | 4 | 0 | 0 | 0 | 0 | 0.32 | 0.016 |
| 35 | WBPH | T65 | 20 | 3 | 0 | 2 | 7 | 1 | 0 | 0 | 0 | 0 | 0.29 | 0.012 |
| 35 | WBPH | T65 | 20 | 4 | 0 | 0 | 1 | 5 | 0 | 0 | 0 | 0 | 0.17 | 0.028 |
| 35 | WBPH | T65 | 20 | 5 | 0 | 1 | 5 | 0 | 0 | 0 | 0 | 0 | 0.17 | 0.023 |
| 35 | WBPH | T65 | 25 | 1 | 0 | 0 | 3 | 1 | 0 | 0 | 0 | 0 | 0.15 | 0.025 |
| 35 | WBPH | T65 | 25 | 2 | 0 | 2 | 4 | 0 | 0 | 0 | 0 | 0 | 0.20 | 0.021 |
| 35 | WBPH | T65 | 25 | 3 | 0 | 2 | 5 | 2 | 0 | 0 | 0 | 0 | 0.33 | 0.024 |
| 35 | WBPH | T65 | 25 | 4 | 0 | 0 | 5 | 1 | 0 | 0 | 0 | 0 | 0.21 | 0.015 |
| 35 | WBPH | T65 | 25 | 5 | 0 | 1 | 2 | 0 | 0 | 0 | 0 | 0 | 0.06 | 0.022 |
