# Supplementary material for: Intraspecific competition counters the effects of elevated and optimal temperatures on phloem-feeding insects in tropical and temperate rice
Source: PLoS One. 2020 Oct 6;15(10):e0240130. doi: 10.1371/journal.pone.0240130 (PMC7538200; doi:10.1371/journal.pone.0240130)
Supplement: S1 Fig — (DOCX) [file pone.0240130.s013.docx]

**Fig. S1. Seedling weight loss per adult planthopper during oviposition experiments.** The loss of dry weight per adult BPH (A,B) and WBPH (C,D) on IR22 (A,C) and T65 (B,D) rice plants are indicated for planthopper densities ranging from 1 to 12 adults per plant. Standard errors are indicated (N = 5)(see also Fig. 4). BPH and WBPH both caused greater reductions in plant biomass at 25 and 30°C compared to 35°C (BPH: F_2,168_ = 67.643, P < 0.001; WBPH: F_2,168_ = 62.348, P < 0.001) and at densities of > 4 per plant (BPH: F_6,168_ = 52.538, P < 0.001; WBPH: F_6,168_ = 36.534, P < 0.001). Similar weight losses across all temperatures at densities of 8-12 adults produced a significant [temperature*density] interaction (BPH: F_12,168_ = 11.454, P < 0.001; WBPH: F_12,168_ = 10.861, P < 0.001).
