# Supplementary material for: Intraspecific competition counters the effects of elevated and optimal temperatures on phloem-feeding insects in tropical and temperate rice
Source: PLoS One. 2020 Oct 6;15(10):e0240130. doi: 10.1371/journal.pone.0240130 (PMC7538200; doi:10.1371/journal.pone.0240130)
Supplement: S2 Fig — (DOCX) [file pone.0240130.s014.docx]

**Fig. S2. Seedling weight loss per nymph weight during experiments.** The loss of dry weight per mg of BPH (A,B) and WBPH (C,D) nymphs on IR22 (A,C) and T65 (B,D) rice plants are indicated for nymph densities ranging from 5 to 25 per plant. Standard errors are indicated (N = 5)(see also Fig. 5). Data for 35°C were excluded from analyses because of low survival and extreme low weights of survivors at that temperature. Nymphs of both species caused greater reductions in plant biomass per unit nymph biomass at 30°C compared to 25°C (BPH: F_1,80_ = 5.596, P = 0.020; WBPH: F_1,80_ = 5.278, P = 0.024) and at densities of ≥ 10 per plant (BPH: F_4,80_ = 8.462, P < 0.001; WBPH: F_4,80_ = 2.431, P = 0.054).
